# Diversity of *Trichoderma* species associated with soil in the Zoige alpine wetland of Southwest China

**DOI:** 10.1038/s41598-022-25223-0

**Published:** 2022-12-15

**Authors:** Gui-Ting Tang, Ying Li, You Zhou, Yu-Hang Zhu, Xiao-Juan Zheng, Xiao-Li Chang, Shi-Rong Zhang, Guo-Shu Gong

**Affiliations:** 1 College of Agronomy, Sichuan Agricultural University, Chengdu, 611130 China; 2 Southeast Chongqing Academy of Agricultural Sciences, Fuling, 408099 China; 3 Environment and Plant Protection Institute, Chinese Academy of Tropical Agricultural Sciences, Haikou, 571101 China; 4 College of Environment, Sichuan Agricultural University, Chengdu, 611130 China

**Keywords:** Fungal genetics, Biodiversity, Eukaryote

## Abstract

The ecology of soil fungi is poorly understood, and recent comprehensive reports on *Trichoderma* are unavailable for any region, including the Zoige alpine wetland ecological region in China. One hundred soil samples were collected from different soil types and soil layers in Zoige alpine wetland ecological regions. Using the traditional suspension plating method, 80 *Trichoderma* strains were chosen to analyze species diversity. After a preliminary classification of morphological characteristics and the genes glyceraldehyde-3-phosphate dehydrogenase (*gpd*), 57 representative strains were selected and eventually identified as seven species via phylogenetic analyses of multilocus sequences based on the genes transcription elongation factor 1 alpha (*tef1*), encoding RNA polymerase II subunit B (*rpb2*) and ATP citrate lyase (*acl1*). Among them, *T. harzianum* was the dominant species isolated from five soil layers and four soil types, and had the highest isolation frequency (23%) in this zone, while *T. polysporum* and *T. pyramidale* were rare species, with isolation frequencies of less than 1%. Our detailed morphological observation and molecular phylogenetic analyses support the recognition of *Trichoderma zoigense* was described for the first time as a new species, while *T. atrobrunneum* as a new record for China was found. Our results will be used as a reference for a greater understanding of soil microbial resources, ecological rehabilitation and reconstructions in the Zoige alpine wetland.

## Introduction

As an essential member of the soil microflora, soil fungi (along with other microorganisms) participate in the material cycle and energy flow in ecosystems. Fungi play an especially vital role in organic decomposition, carbon and nitrogen storage, biogeochemical cycles, soil stabilization, and plant parasitism^1–5^, and fungal diversity has been recognized as a critical indicator of soil health^6,7^. Research on the soil ecological environment, especially on the diversity of fungi in some important ecological regions, has recently gained much attention. More specifically, the role of soil microorganisms in promoting the regulatory mechanism of plant communities has become increasingly recognized. Thus, the microbial diversity on the surface and subsurface has remained a significant theme on recent ecological research^8^. For instance, in China, fungal flora and soil diversity have been reported in the Changbai Mountains, three nature reserves in Jiuzhaigou County, and Mount Gongga^9,10^.

The genus *Trichoderma*, which includes more than 200 species in various geographical regions and climatic zones around the world, a number that is regularly increasing^11–13^, is the most common fungi in soil and rotting wood^14,15^. *Trichoderma* have a fine metabolic regulation that is able to respond to environmental changes and to nutrient and oxygen limitations. They therefore produce a range of enzymes to degrade homopolysaccharides and heteropolysaccharides, which are important carbon sink ecosystem. Some species of *Trichoderma* have a powerful phosphate-solubilizing ability, whereas other species act as industrial enzymes for the preparation of cellulose, hemicellulase, xylanase, chitinase, protease, and antibiotics in agricultural production^16–22^. In addition, the genus has been confirmed to be associated with the ability to control plant pathogens, promote plant growth, stimulate plant immunity and remediate soil contaminants^23–26^.

*Hypocrea* and *Trichoderma* were once treated as two separate genera, although studies by the Tulasne brothers indicated that *Hypocrea* is a sexual morph (teleomorph) of *Trichoderma.* Combining *Hypocrea* and *Trichoderma*, Doi et al.^27–38^ summarized previously reported species and revised nearly 50 new species of the genus based primarily on morphological characteristics. However, distinguishing *Trichoderma* species using traditional morphological methods is difficult and inaccurate^39^. Due to the overlap between intraspecific and interspecific sequence differences in the nuclear rDNA ITS region, it is not suitable for multiple gene identification. Multiple molecular techniques have been applied for identifying *Trichoderma*; for example the genes encoding RNA polymerase II subunit B (*rpb2*), transcription elongation factor 1 alpha (*tef1*) and ATP citrate lyase (*acl1*) have commonly been used either individually or in combination^40–47^. A combination of phylogenetic analyses of multiple genes and morphological characteristics has been widely used to study fungal diversity, and nearly a hundred new species of this genus have been recorded in various ecological zones worldwide^40,46–60^.

Wetlands represent a significant land resource and a source of natural resources with various functions, such as forest, cultivated land and sea. These areas are rich in biological diversity in terms of both the ecological landscape and the human living environment. The Zoige alpine wetland is one of the most important wetlands in China because of its complex natural environment, abundant ecological resources, and unique climatic conditions. Although reports have addressed the local soil active organic carbon, vegetation, animal community, gas flux, functional bacteria and microorganism methanogens^61–68^, the ecology of soil fungi is poorly understood, and recent comprehensive reports on *Trichoderma* are not available for any region, including the Zoige alpine wetland ecological region in China. Only Feng et al.^69^ have analyzed the fungal community structure in the soil of this region via a combination of BIOLOG analysis and traditional cultural methods. Because morphological and molecular tools are ideal for assessments of the species diversity in all geographical regions, the work described here was designed to investigate the species diversity of the genus *Trichoderma* in the unique ecological environment of the Zoige alpine wetland, with an emphasis on four major soil types (peat soil, meadow soil, subalpine meadow soil, and aeolian sandy soil). Our results may be used as a reference for a greater understanding of soil microorganisms in various ecological regions, ecological rehabilitation and reconstruction, and microbial resources.

## Results

### *Trichoderma* species collection

Eighty strains were obtained from 100 soil samples collected from Zoige alpine wetland ecological regions in China. Details of the strains isolated from soil samples are given in Table 1. All strains were subsequently used for morphological identification, while fifty-seven were used for phylogenetic analysis.

**Table 1:** Details of 80 *Trichoderma* isolates from the Zoige alpine wetland in this study.

Isolates	Geographical location	Altitude (m a.s.l.)	Soil types	Soil layers (m)	Species
T1	102° 29′ 05.8″, 33° 43′ 17.7″	3448	Aeolian sand soil	0–10	*Trichoderma harzianum*
T2	102° 56′ 26.3″, 33° 36′ 13.4″	3446	Peat soil	0–10	*T. harzianum*
T3	102° 42′ 52.1″, 33° 31′ 18.5″	3461	Subalpine meadow soil	10–20	*T. harzianum*
T4	102° 29′ 05.8″, 33° 43′ 17.7″	3448	Aeolian sand soil	0–10	*T. harzianum*
T5	102° 29′ 05.8″, 33° 43′ 17.7″	3448	Aeolian sand soil	0–10	*T. harzianum*
T6	102° 29′ 05.8″, 33° 43′ 17.7″	3448	Aeolian sand soil	0–10	*T. harzianum*
T7	102° 56′ 26.3″, 33° 36′ 13.4″	3446	Peat soil	0–10	*T. harzianum*
T8	102° 55′ 18.5″, 33° 35′ 36.0″	3462	Peat soil	0–10	*T. harzianum*
T9	102° 29′ 04.6″, 33° 43′ 17.6″	3450	Aeolian sand soil	0–10	*T. harzianum*
T10	102° 55′ 20.3″, 33° 37′ 07.8″	3443	Peat soil	0–10	*T. harzianum*
T11	102° 32′ 25.4″, 33° 45′ 55.8″	3488	Peat soil	0–10	*T. harzianum*
T12	102° 29′ 04.6″, 33° 43′ 17.6″	3450	Aeolian sand soil	0–10	*T. harzianum*
T13	102° 49′ 44.4″, 33° 38′ 01.1″	3446	Meadow soil	0–10	*T. harzianum*
T14	102° 56′ 37.3″, 33° 35′ 12.8″	3435	Peat soil	0–10	*T. harzianum*
T15	102° 42′ 52.1″, 33° 31′ 18.5″	3461	Subalpine meadow soil	20–30	*T. harzianum*
T16	102° 29′ 57.5″, 33° 23′ 56.1″	3452	Meadow soil	0–10	*T. alni*
T17	102° 55′ 18.5″, 33° 35′ 36.0″	3462	Peat soil	0–10	*T. harzianum*
T18	102° 49′ 44.4″, 33° 38′ 01.1″	3446	Meadow soil	0–10	*T. harzianum*
T19	102° 55′ 18.5″, 33° 35′ 36.0″	3462	Peat soil	0–10	*T. harzianum*
T20	102° 55′ 18.5″, 33° 35′ 36.0″	3462	Peat soil	0–10	*T. pyramidale*
T21	102° 56′ 26.3″, 33° 36′ 13.4″	3446	Peat soil	0–10	*T. harzianum*
T22	102° 56′ 37.3″, 33° 35′ 12.8″	3435	Peat soil	0–10	*T. harzianum*
T23	102°49′44.4″, 33° 38′ 01.1″	3446	Meadow soil	10–20	*T. harzianum*
T24	102° 37′ 27.9″, 33° 50′ 31.1″	3433	Subalpine meadow soil	0–10	*T. alni *
T25	102° 29′ 57.5″, 33° 23′ 56.1″	3452	Meadow soil	0–10	*T. zoigense *
T26	102° 32′ 25.4″, 33° 45′ 55.8″	3488	Peat soil	0–10	*T. harzianum *
T27	102° 49′ 44.4″, 33°38′ 01.1″	3446	Meadow soil	0–10	*T. rossicum *
T28	102° 29′ 57.5″, 33° 23′ 56.1″	3452	Meadow soil	0–10	*T. alni *
T29	102° 42′ 52.1″, 33° 31′ 18.5″	3461	Subalpine meadow soil	0–10	*T. harzianum *
T30	102° 42′ 52.1″, 33° 31′ 18.5″	3461	Subalpine meadow soil	0–10	*T. harzianum *
T31	102° 42′ 52.1″, 33° 31′ 18.5″	3461	Subalpine meadow soil	0–10	*T. harzianum *
T32	102° 29′ 04.6″, 33° 43′ 17.6″	3450	Aeolian sand soil	0–10	*T. harzianum *
T33	102° 29′ 05.8″, 33° 43′ 17.7″	3448	Aeolian sand soil	0–10	*T. harzianum *
T34	102° 32′ 25.4″, 33° 45′ 55.8″	3488	Peat soil	0–10	*T. harzianum *
T35	102° 51′ 22.1″, 33° 32′ 24.6″	3488	Peat soil	0–10	*T. harzianum *
T36	102° 55′ 18.5″, 33° 35′ 36.0″	3462	Peat soil	0–10	*T. alni *
T37	102° 55′ 18.5″, 33° 35′ 36.0″	3462	Peat soil	0–10	*T. harzianum *
T38	102° 56′ 26.3″, 33° 36′ 13.4″	3446	Peat soil	0–10	*T. harzianum *
T39	102° 37′ 03.3″, 33° 57′ 33.3″	3437	Peat soil	0–10	*T. atrobrunneum *
T40	102° 56′ 57.9″, 33° 36′ 29.8″	3493	Subalpine meadow soil	0–10	*T. alni *
T41	102° 29′ 09.9″, 33° 26′ 47.9″	3452	Subalpine meadow soil	0–10	*T. alni *
T42	102° 29′ 26.9″, 33° 43′ 14.3″	3462	Aeolian sand soil	0–10	*T. atrobrunneum *
T43	102° 29′ 57.5″, 33° 23′ 56.1″	3452	Meadow soil	0–10	*T. zoigense *
T44	102° 29′ 57.5″, 33° 23′ 56.1″	3452	Meadow soil	20–30	*T. zoigense *
T45	102° 49′ 44.4″, 33° 38′ 01.1″	3446	Meadow soil	20–30	*T. harzianum *
T46	102° 42′ 52.1″, 33° 31′ 18.5″	3461	Subalpine meadow soil	0–10	*T. harzianum *
T47	102° 42′ 52.1″, 33° 31′ 18.5″	3461	Subalpine meadow soil	0–10	*T. harzianum *
T48	102° 52′ 33.1″, 33° 33′ 55.9″	3501	Subalpine meadow soil	0–10	*T. zoigense *
T49	102° 52′ 33.1″, 33° 33′ 55.9″	3501	Subalpine meadow soil	10–20	*T. harzianum *
T50	102° 32′ 25.4″, 33° 45′ 55.8″	3488	Peat soil	50–100	*T. polysporum *
T51	102° 33′ 21.5″, 33° 54′ 57.6″	3426	Subalpine meadow soil	30–50	*T. rossicum *
T52	102° 33′ 21.5″, 33° 54′ 57.6″	3426	Subalpine meadow soil	30–50	*T. rossicum *
T53	102° 55′ 18.5″, 33° 35′ 36.0″	3462	Peat soil	0–10	*T. alni *
T54	102° 29′ 09.7″, 33° 28′ 02.6″	3480	Subalpine meadow soil	0–10	*T. alni *
T55	102° 55′ 18.5″, 33° 35′ 36.0″	3462	Peat soil	0–10	*T. harzianum *
T56	102° 42′ 52.1″, 33° 31′ 18.5″	3461	Subalpine meadow soil	0–10	*T. harzianum *
T57	102° 37′ 03.3″, 33° 57′ 33.3″	3437	Peat soil	0–10	*T. atrobrunneum *
T58	102° 29′ 57.5″, 33° 23′ 56.1″	3452	Meadow soil	0–10	*T. zoigense *
T59	102° 56′ 37.3″, 33° 35′ 12.8″	3435	Peat soil	0–10	*T. harzianum *
T60	102° 29′ 57.5″, 33° 23′ 56.1″	3452	Meadow soil	0–10	*T. zoigense *
T61	102° 29′ 09.9″, 33° 26′ 47.9″	3452	Subalpine meadow soil	0–10	*T. alni *
T62	102° 56′ 57.9″, 33° 36′ 29.8″	3493	Subalpine meadow soil	0–10	*T. alni *
T63	102° 37′ 03.3″, 33° 57′ 33.3″	3437	Peat soil	0–10	*T. harzianum *
T64	102° 49′ 44.4″, 33° 38′ 01.1″	3446	Meadow soil	0–10	*T. rossicum *
T65	102° 52′ 33.1″, 33° 33′ 55.9″	3501	Subalpine meadow soil	0–10	*T. harzianum *
T66	102° 29′ 57.5″, 33° 23′ 56.1″	3452	Meadow soil	0–10	*T. zoigense *
T67	102° 56′ 57.9″, 33° 36′ 29.8″	3493	Subalpine meadow soil	0–10	*T. alni *
T68	102° 36′ 51.0″, 33° 26′ 10.7″	3531	Subalpine meadow soil	0–10	*T. alni *
T69	102° 37′ 12.6″, 33° 51′ 02.1″	3434	Subalpine meadow soil	0–10	*T. alni *
T70	102° 56′ 57.9″, 33° 36′ 29.8″	3493	Subalpine meadow soil	0–10	*T. alni *
T71	102° 29′ 57.5″, 33° 23′ 56.1″	3452	Meadow soil	0–10	*T. zoigense *
T72	102° 29′ 57.5″, 33° 23′ 56.1″	3452	Meadow soil	10–20	*T. alni *
T73	102° 29′ 05.8″, 33° 43′ 17.7″	3448	Aeolian sand soil	0–10	*T. harzianum *
T74	102° 29′ 05.8″, 33° 43′ 17.7″	3448	Aeolian sand soil	0–10	*T. harzianum *
T75	102° 37′ 12.6″, 33° 51′ 02.1″	3426	Subalpine meadow soil	30–50	*T. harzianum *
T76	102° 49′ 44.4″, 33° 38′ 01.1″	3446	Meadow soil	30–50	*T. harzianum *
T77	102° 29′ 57.5″, 33° 23′ 56.1″	3452	Meadow soil	50–100	*T. harzianum *
T78	102° 51′ 22.1″, 33° 32′ 24.6″	3444	Peat soil	30–50	*T. harzianum *
T79	102° 32′ 25.4″, 33° 45′ 55.8″	3488	Peat soil	50–100	*T. harzianum *
T80	102° 54′ 15.2″, 33° 34′ 72.2″	3449	Peat soil	0–10	*T. harzianum *

### Phylogenetic analysis

The ITS region used preliminarily as a species identification criterion was applied to TrichOKey at www.ISTH.info^70^. However, the ITS region has a low number of variable sites and long insertions in certain species; thus, it is unsuitable for a phylogenetic reconstruction of this group^41^. Our study successfully amplified most fragments of the genes *tef1*, *rpb2*, and *acl1*. We also designed a pair of new primers based on the full-length tef1 gene, 5′-GAGAAGTTCGAGAAGGTGAGC-3′ and 5′-ATGTCACGGACGGCGAAAC-3′, with which a 1.4-kb fragment was amplified for most isolates.

**Figure 1:**
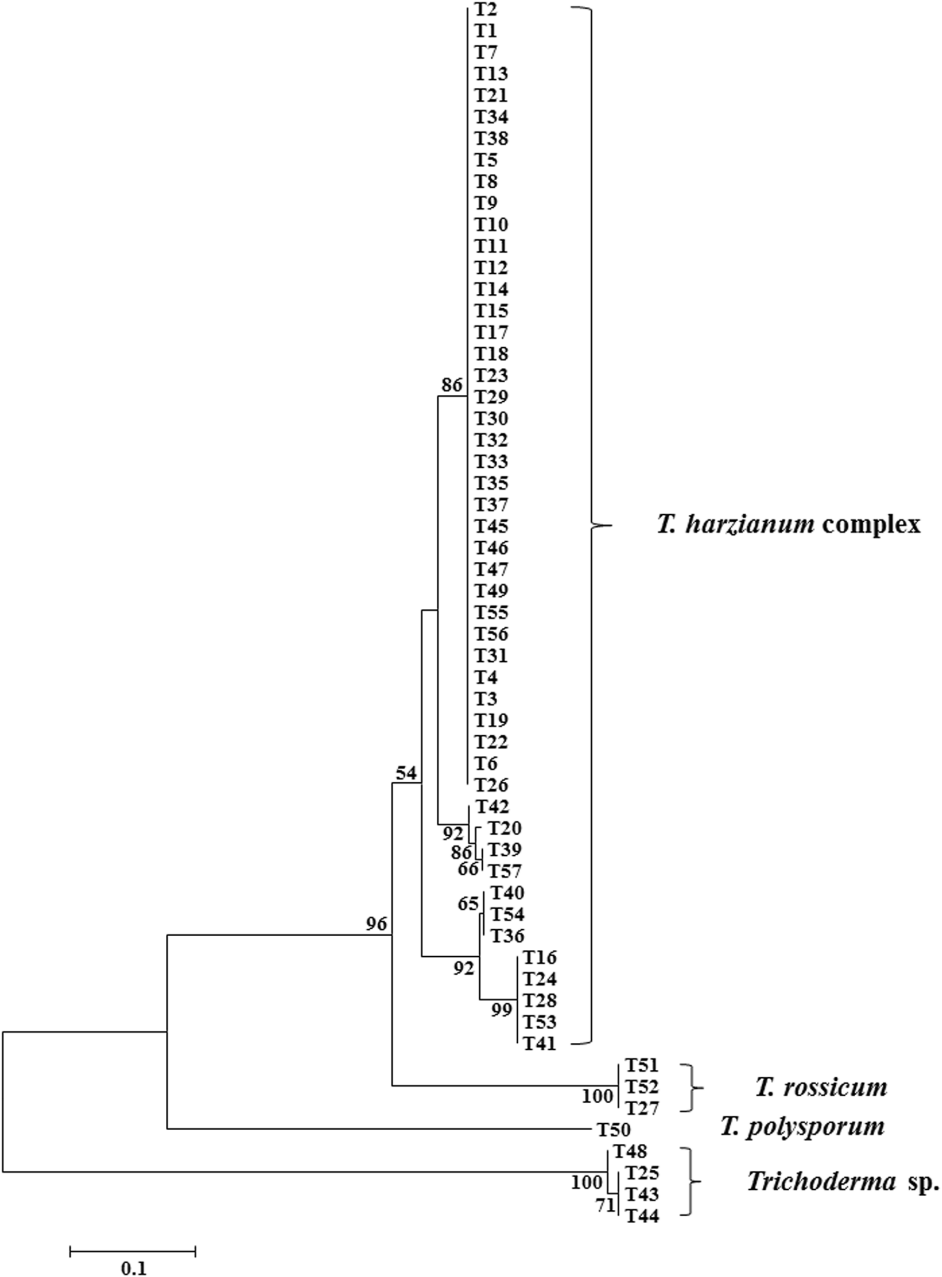
Neighbor-joining tree based on partial gpd gene sequences from 57 *Trichoderma* isolates. Parsimony bootstrap values of more than 50% are shown at nodes.

**Figure 2:**
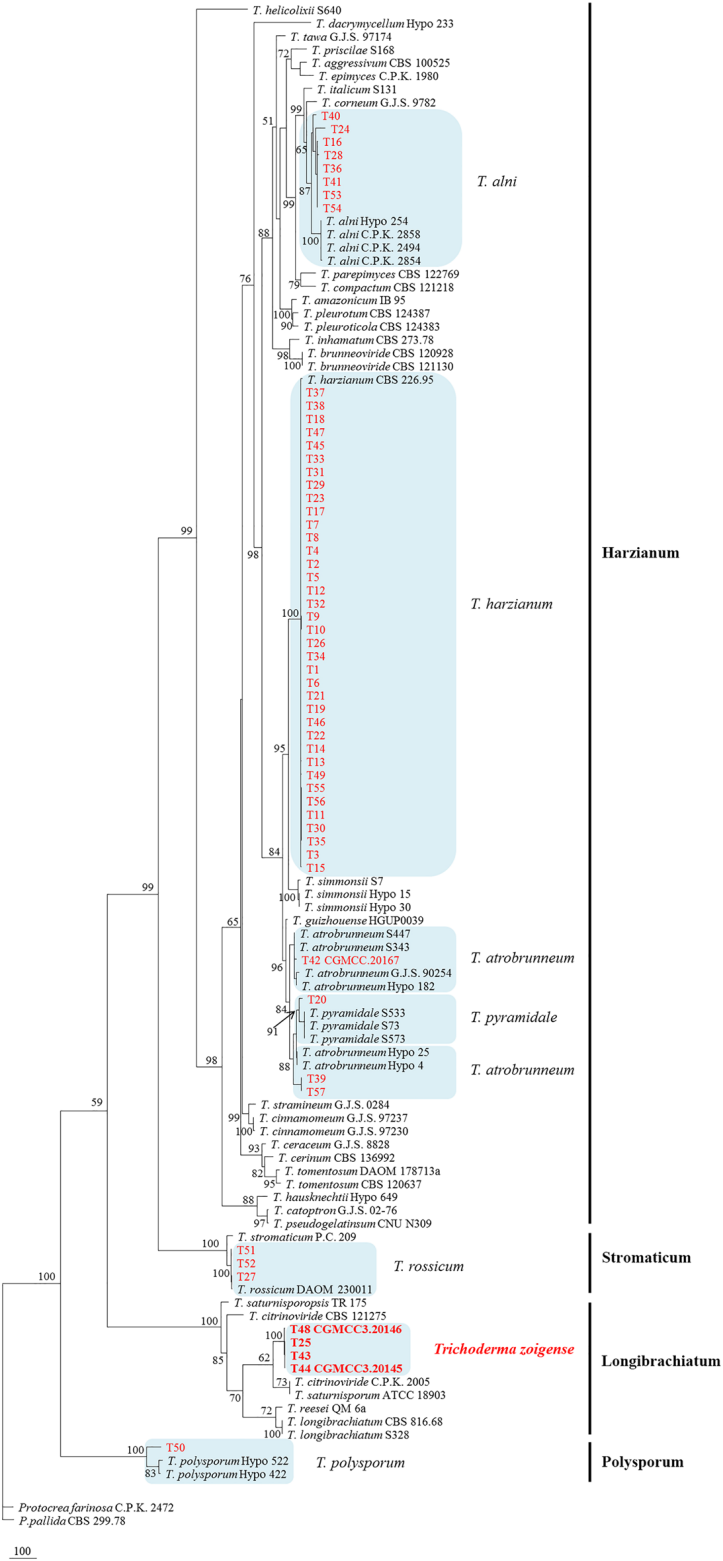
Maximum parsimony tree of *Trichoderma* species inferred from the combined *rpb2*, *tef1* and *acl1* partial sequences. Maximum parsimony bootstrap values above 50% are shown at nodes. The tree was rooted with *Protocrea farinose* and *P. pallida* Isolates from this study are shown in red (new species in bold).

All samples analyzed in our study were divided into 4 primary clades based on the gpd gene region, including 49 strains from the *T. harzianum* complex, 3 *T. rossicum* strains, 1 *T. polysporum* strain and one unknown species (4 *Trichoderma* sp. strains) (Fig. 1). Maximum parsimony analysis was conducted among 101 strains, with *Protocrea farinosa* (CPK 2472) and *P. pallida* (CBS 299.78) used as outgroup (Table 2). The dataset for the *rpb2*, *tef1* and *acl1* genes contained 3403 characteristics, among which 1152 were parsimony-informative, 988 were variable and parsimony-uninformative, and 1263 were constant. The most parsimonious trees are shown in Fig. 2 (tree length = 5054, consistency index = 0.6005, homoplasy index = 0.3995, retention index = 0.8105, rescaled consistency index = 0.4867).

**Table 2:** *Trichoderma* strain included in the multi-gene sequence analysis, with details of clade, strain number, location, and GenBank accessions of the sequences generated.

Species	Clade	Strain	Location	ITS	TEF	RPB2	ACL1	GPD
*Trichoderma aggressivum *	Green/harzianum	CBS 100525	UK: England	–	AF534614	AF545541	–	–
*T. alni *	Green/harzianum	Hypo 254 = CBS 120633 (T)	UK: England	EU518651	EU498312	EU498349	KJ664942	–
		C.P.K. 2494	–	–	EU498313	EU498350	–	–
		C.P.K. 2854	–	–	EU498314	EU498351	–	–
		C.P.K. 2858	–	–	EU498315	–	–	–
		T16	China	KX632517	KX632574	KX632631	KX632688	KX632745
		T24	China	KX632518	KX632575	KX632632	KX632689	KX632746
		T28	China	KX632519	KX632576	KX632633	KX632690	KX632747
		T36	China	KX632520	KX632577	KX632634	KX632691	KX632748
		T40	China	KX632521	KX632578	KX632635	KX632692	KX632749
		T41	China	KX632522	KX632579	KX632636	KX632693	KX632750
		T53	China	KX632523	KX632580	KX632637	KX632694	KX632751
		T54	China	KX632524	KX632581	KX632638	KX632695	KX632752
*T. amazonicum *	Green/harzianum	IB 95	Peru	–	HM142377	HM142368	–	–
*T. atrobrunneum *	Green/harzianum	G.J.S. 90-254	Germany	–	AF443943	FJ442735	KJ664942	–
		Hypo 25	Austria	–	KJ665359	–	–	–
		S343	Spain	–	KJ665383	–	–	–
		S447	Spain	–	KJ665396	–	–	–
		Hypo 4	Germany	–	KJ665365	–	KJ664949	–
		Hypo 182	Germany	–	KJ665357	–	KJ664948	–
		T39	China	KX632514	KX632571	KX632628	KX632685	KX632742
		T42(CGMCC 3.20167)	China	KX632515	KX632572	KX632629	KX632686	KX632743
		T57	China	KX632516	KX632573	KX632630	KX632687	KX632744
*T. brunneoviride *	Green/harzianum	CBS 120928	Austria	EU518661	EU498318	EU498358	–	–
		CBS 121130 (T)	Germany	–	EU498316	–	–	–
*T. catoptron *	Green/harzianum	G.J.S. 02-76	Sri Lanka	AY737766	AY391963	AY391900	KJ664969	–
*T. ceraceum *	Green/harzianum	G.J.S. 88-26	USA	–	AY391964	AY391901	–	–
*T. cerinum *	Green/harzianum	CBS 136992 = S357	France	–	KF134797	KF134788	KJ664977	–
*T. cinnamomeum *	Green/harzianum	G.J.S. 97-230 = CBS 114235 (T)	USA	–	–	AY391918	KJ664965	–
		G.J.S. 97-237	USA	AY737759	AY391979	AY391920	–	–
*T. citrinoviride *	Longibrachiatum	CBS 121275 = Hypo 162	Germany	–	–	FJ860586	KJ64978	–
		C.P.K. 2005	Austria	–	FJ860694	–	–	–
*T. compactum *	Green/harzianum	CBS 121218 (T)	China	–	KF134798	KF134789	KJ664984	–
*T. corneum *	Green/harzianum	G.J.S. 97-82	Thailand	–	KJ665455	KJ665252	KJ664985	–
*T. dacrymycellum *	Green/harzianum	Hypo 233 = WU 29044	Germany	FJ860749	FJ860633	FJ860533	KJ664993	–
*T. epimyces *	Green/harzianum	C.P.K. 1980	Germany	EU518662	EU498319	EU498359	KJ664993	–
*T. guizhouense *	Green/harzianum	HGUP0039	China	–	JX089585	–	–	–
*T. harzianum *	Green/harzianum	CBS 226.95 (T neo)	UK: England	AY605713	AF534621	AF545549	–	–
		T1	China	KX632476	KX632533	KX632590	KX632647	KX632704
		T2	China	KX632477	KX632534	KX632591	KX632648	KX632705
		T3	China	KX632478	KX632535	KX632592	KX632649	KX632706
		T4	China	KX632479	KX632536	KX632593	KX632650	KX632707
		T5	China	KX632480	KX632537	KX632594	KX632651	KX632708
		T6	China	KX632481	KX632538	KX632595	KX632652	KX632709
		T7	China	KX632482	KX632539	KX632596	KX632653	KX632710
		T8	China	KX632483	KX632540	KX632597	KX632654	KX632711
		T9	China	KX632484	KX632541	KX632598	KX632655	KX632712
		T10	China	KX632485	KX632542	KX632599	KX632656	KX632713
		T11	China	KX632486	KX632543	KX632600	KX632657	KX632714
		T12	China	KX632487	KX632544	KX632601	KX632658	KX632715
		T13	China	KX632488	KX632545	KX632602	KX632659	KX632716
		T14	China	KX632489	KX632546	KX632603	KX632660	KX632717
		T15	China	KX632490	KX632547	KX632604	KX632661	KX632718
		T17	China	KX632491	KX632548	KX632605	KX632662	KX632719
		T18	China	KX632492	KX632549	KX632606	KX632663	KX632720
		T19	China	KX632493	KX632550	KX632607	KX632664	KX632721
		T21	China	KX632494	KX632551	KX632608	KX632665	KX632722
		T22	China	KX632495	KX632552	KX632609	KX632666	KX632723
		T23	China	KX632496	KX632553	KX632610	KX632667	KX632724
		T26	China	KX632497	KX632554	KX632611	KX632668	KX632725
		T29	China	KX632498	KX632555	KX632612	KX632669	KX632726
		T30	China	KX632499	KX632556	KX632613	KX632670	KX632727
		T31	China	KX632500	KX632557	KX632614	KX632671	KX632728
		T32	China	KX632501	KX632558	KX632615	KX632672	KX632729
		T33	China	KX632502	KX632559	KX632616	KX632673	KX632730
		T34	China	KX632503	KX632560	KX632617	KX632674	KX632731
		T35	China	KX632504	KX632561	KX632618	KX632675	KX632732
		T37	China	KX632505	KX632562	KX632619	KX632676	KX632733
		T38	China	KX632506	KX632563	KX632620	KX632677	KX632734
		T45	China	KX632507	KX632564	KX632621	KX632678	KX632735
		T46	China	KX632508	KX632565	KX632622	KX632679	KX632736
		T47	China	KX632509	KX632566	KX632623	KX632680	KX632737
		T49	China	KX632510	KX632567	KX632624	KX632681	KX632738
		T55	China	KX632511	KX632568	KX632625	KX632682	KX632739
		T56	China	KX632512	KX632569	KX632626	KX632683	KX632740
*T. hausknechtii *	Green/harzianum	Hypo 649 = CBS 133493 (T)	France	–	KJ665515	KJ665276	KJ665034	–
*T. helicolixii *	Green/harzianum	S640 = CBS 133499 (T)	Greece	–	KJ665517	KJ665278	KJ665036	–
*T. inhamatum *	Green/harzianum	CBS 273.78 (T)	Colombia	–	AF348099	FJ442725	–	–
*T. italicum *	Green/harzianum	S131 = CBS 132567 (T)	Italy	–	KJ665525	KJ665282	KJ665045	–
*T. longibrachiatum *	Longibrachiatum	CBS 816.68	USA	–	EU401591	DQ087242	–	–
		S328	Spain	–	JQ685867	JQ685883	–	–
*T. parepimyces *	Green/harzianum	CBS 122769 (T)	Austria	–	FJ860664	FJ860562	KJ665138	–
*T. pleuroti *	Green/harzianum	CBS 124387 (T)	Korea	–	HM142382	HM142372	–	–
*T. pleuroticola *	Green/harzianum	CBS 124383 (T)	Korea	–	HM142381	HM142371	–	–
*T. polysporum *	Polysporum	Hypo 422 = C.P.K. 2461	Austria	–	–	FJ179613	KJ665057	–
		Hypo 522 = C.P.K. 3131	Austria	–	FJ860661	JQ685878	KJ665138	–
		T50	China	KX632525	KX632582	KX632639	KX632696	KX632753
*T. priscilae *	Green/harzianum	S168 = CBS 131487 (T)	Spain	–	KJ665691	KJ665333	KJ665151	–
*T. pseudogelatinsum *	Green/harzianum	CNU N309	Korea	–	HM920202	HM920173	–	–
*T. pyramidale *	Green/harzianum	S73 = CBS 135574 (T)	Italy	–	KJ665699	KJ665334	KJ665116	–
		S573	Italy	–	KJ665698	–	–	–
		S533	Spain	–	KJ665697	–	KJ665162	–
		T20	China	KX632513	KX632570	KX632627	KX632684	KX632741
*T. reesei *	Longibrachiatum	QM 6a = CBS 383.78 (T)	New Guinea	–	–	HM182969	KJ665163	–
*T. rossicum *	Stromaticum	DAOM 230011 (T)	Russia	–	AY937441	HQ342288	–	–
		T27	China	KX632526	KX632583	KX632640	KX632697	KX632754
		T51	China	KX632527	KX632584	KX632641	KX632698	KX632755
		T52	China	KX632528	KX632585	KX632642	KX632699	KX632756
*T. saturnisporopsis *	Longibrachiatum	TR 175 = C.P.K. 1356 (T)	USA	–	–	DQ857348	–	–
*T. saturnisporum *	Longibrachiatum	ATCC 18903 = CBS 330.70	USA	–	EU280044	DQ087243	–	–
*T. simmonsii *	Green/harzianum	Hypo 15 = C.P.K. 1596	Austria	–	KJ665706	–	–	–
		Hypo 30 = C.P.K. 2391	Austria	–	KJ665707	–	KJ665182	–
		S7	Italy	–	KJ665719	KJ665337	KJ665182	–
*T. stramineum *	Green/harzianum	G.J.S.02-84 = CBS 114248	Sri Lanka	AY737765	AY391999	AY391945	–	–
*T. stromaticum *	Stromaticum	P.C. 209	Brazil	–	AF534613	AF545539	KJ665185	–
*T. tawa *	Green/harzianum	G.J.S. 97-174	Thailand	AY737756	AY392004	AY391956	–	–
*T. tomentosum *	Green/harzianum	CBS 120637	Austria	FJ860744	FJ860629	FJ860532	KJ665222	–
		DAOM 178713a (T)	Canada	–	AF534630	AF545557	–	–
*T. zoigense *	Longibrachiatum	T25	China	KX632529	KX632586	KX632643	KX632700	KX632757
		T43	China	KX632530	KX632587	KX632644	KX632701	KX632758
		T44 (CGMCC 3.20145)	China	KX632531	KX632588	KX632645	KX632702	KX632759
		T48 (CGMCC 3.20146)	China	KX632532	KX632589	KX632646	KX632703	KX632760
*Protocrea farinosa *	Outgroup	CBS 121551	Austria	–	–	EU703935	–	–
		C.P.K. 2472	Austria	–	EU703892	–	–	–
*P. pallida *	Outgroup	CBS 121552	Denmark	–	–	EU703944	–	–
		CBS 299.78 (T)	USA	–	EU703900	–	–	–

The phylogram showed that 57 stains belonged to the following four clades: Harzianum, Polysporum, Stromaticum, and Longibrachiatum. The strains of the first three clades with neighboring named species were well supported by bootstrap values greater than 90%. The Harzianum clade contained *T. alni*, *T. atrobrunneum*, *T. harzianum* and *T. pyramidale* of the *Trichoderma* species complex. The Polysporum clade contained only *T. polysporum*, and the Stromaticum clade contained *T. rossicum*. The Longibrachiatum clade contained four strains of *Trichoderma* sp., T25, T43, T44 and T48, which were separated from any other known taxa of this clade showed a low bootstrap value (MPBP = 62%) with *T. citrinoviride* and *T. saturnisporum*. We thus regarded it as a new species and named it *Trichoderma zoigense*, as described in the next section.

### Growth rates

**Figure 3:**
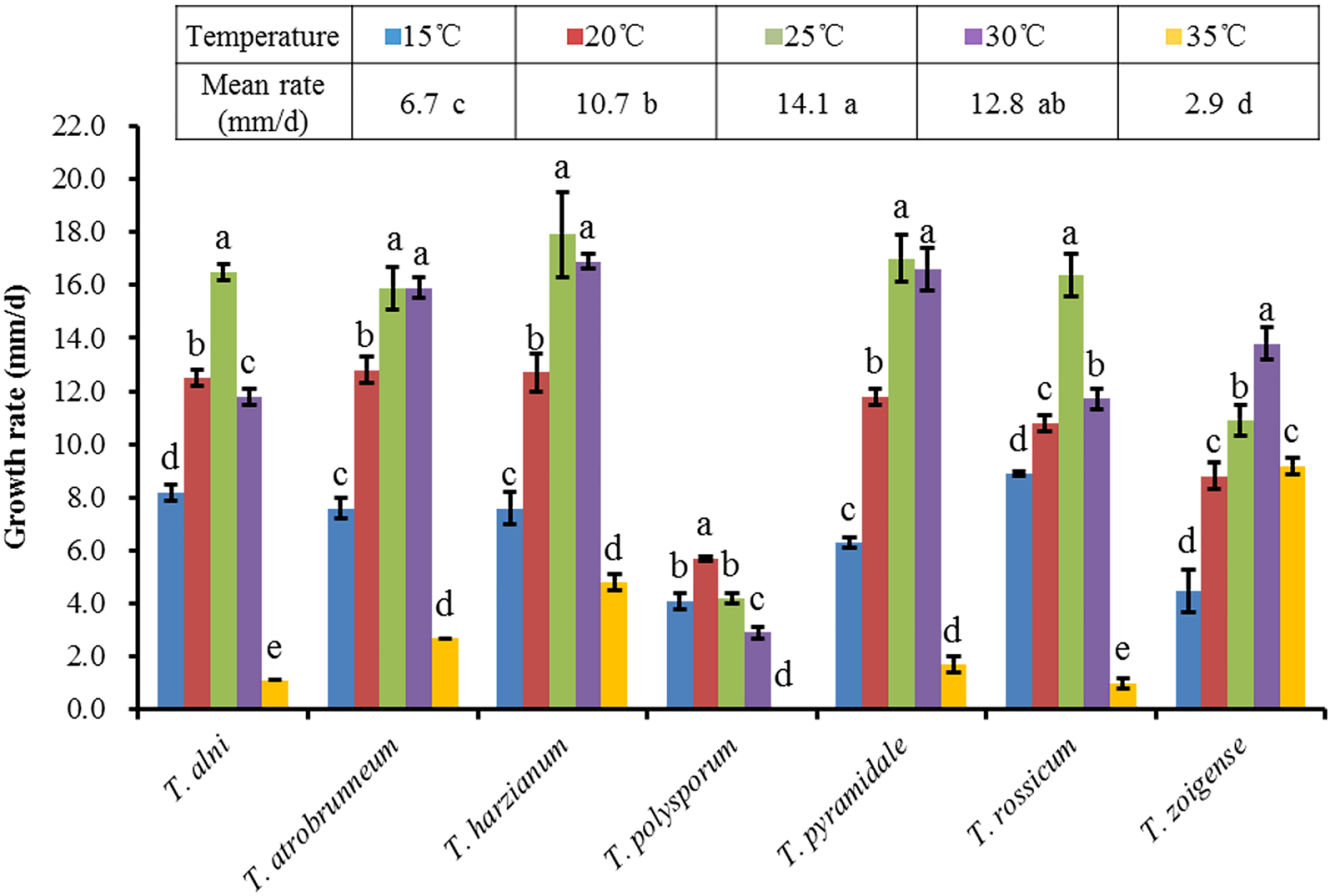
Growth rates of 7 species of *Trichoderma* on CMD given as mm per day at five temperatures. The values were the means of 3–5 experiments, with 1–3 representative isolates per species.

As shown in Fig. 3, the genus *Trichoderma* from Zoige alpine wetland ecological regions was able to grow in a range from 15 to 35 °C, and the suitable growth temperature for most species ranged from 20 to 30 °C. All seven species identified had normal viability at relatively low temperature (15 °C), and they rarely grew well over 35 °C except for *T. zoigense*. For *T. atrobrunneum, T. harzianum and T. pyramidale*, the optimum growth temperature on CMD was 25 to 30 °C. *T. alni* and *T. rossicum* preferred a cool growth environment, with an optimum temperature of 25 °C, whereas *T. zoigense* was more partial to a hot environment, with an optimum temperature of 30 °C, and it even grew well up to 35 °C. *T. polysporum* was the only slow-growing species that grew with less than 6.0 mm/day between 15 and 30 °C and did not survive at 35 °C. The above results showed that all species had different growth rates but were not completely differentiated from each other on CMD. These species were roughly divided into four groups based on their optimum growth temperature.

### Relationship with ecological factors

Our results revealed a substantial disparity in the number and distribution of *Trichoderma* species among Zoige alpine wetland ecological regions (Tables 3, 4). Table 3 showed that *T. harzianum* was found in all four soil types, but most isolates of this species were obtained from peat soil. *T. rossicum*, *T. alni* and *T. zoigense* were also present in meadow soil and subalpine meadow soil, whereas *T. atrobrunneum* was found in aeolian sandy soil and peat soil. *T. polysporum* was found only in peat soil.

**Table 3:** Isolation frequency of *Trichoderma* species in different soil types (%).

Species	Meadow soil	Subalpine meadow soil	Aeolian sand soil	Peat soil	total
*T. harzianum*	5	6	2	10	23
*T. rossicum*	1	1	0	0	2
*T. alni*	2	6	0	1	9
*T. zoigense*	2	1	0	0	3
*T. atrobrunneum*	0	0	1	1	2
*T. polysporum*	0	0	0	1	1
*T. pyramidale*	0	0	0	1	1

**Table 4:** Isolation frequency of *Trichoderma* species in different soil layers (%) species.

	0–10	10–20	20–30	30–50	50–100	Total
*T. harzianum*	13	3	2	3	2	23
*T. rossicum*	1	0	0	1	0	2
*T. alni*	8	1	0	0	0	9
*T. zoigense*	2	0	1	0	0	3
*T. atrobrunneum*	2	0	0	0	0	2
*T. polysporum*	0	0	0	0	1	1
*T. pyramidale*	1	0	0	0	0	1

In regard to the different soil layers shown in Table 4, *T. harzianum* was widely distributed in the five soil layers at depths of 0–100 cm. *T. rossicum*, *T. alni* and *T. zoigense* were isolated mainly from the soil layers at depths of 0–50 cm. Both *T. atrobrunneum* and *T. pyramidale* were isolated from depths of 0–10 cm, and *T. polysporum* was found only in the soil layers at depths of 50–100 cm.

Regarding isolation frequency, *T. harzianum* was the most common of the seven species with a 23% isolation frequency, and it was therefore the dominant species in the zone, while the rare species *T. polysporum* and *T. pyramidale* had the lowest isolation frequencies at 1%.

### Taxonomy

#### New species

**Figure 4:**
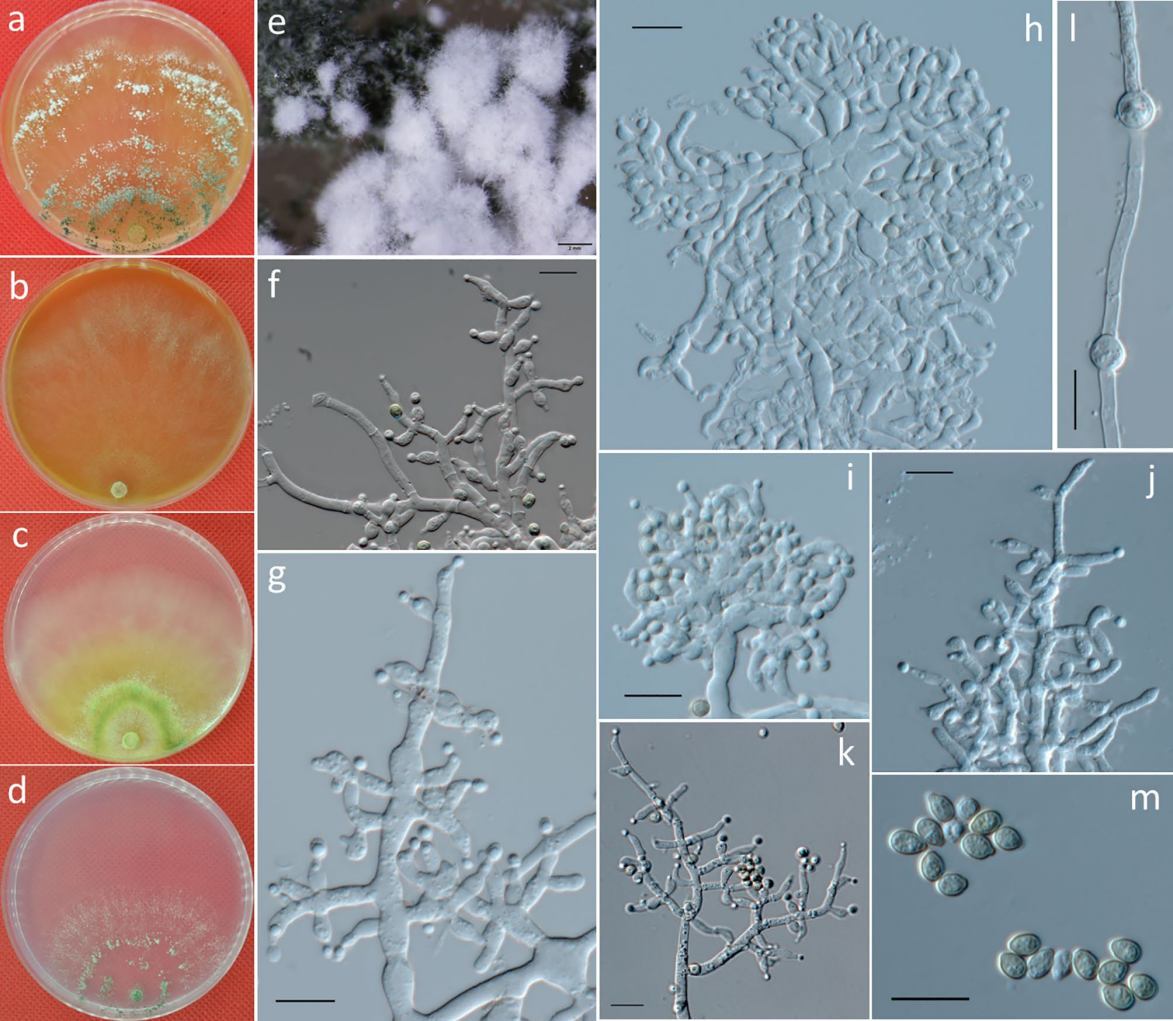
Cultures and asexual morph of *Trichoderma zoigense.* (**a–d**). Cultures at 20 °C [(**a**) on CMD, 7 days; (**b**) on MEA, 4 days; (**c**) on PDA, 4 days; and (**d**) on SNA, 7 days]. (**e**) Conidiation tuft (CMD, 4 days). (**f–k**) Conidiophores and phialides (CMD, 5–7 days). (**l**) Chlamydospores (PDA, 8 days). (**m**) Conidia (CMD, 5 days). Scale bars: (**e**) = 2 mm; (**f–m**) = 10 μm.

***Trichoderma zoigense*** G.S. Gong & G.T. Tang, *sp. nov*. (Fig. 4).

MycoBank: MB 82114.

**Typification:** CHINA. SICHUAN PROVINCE: Zoige Alpine Wetland, on soil, 29 June 2013, G.S. Gong T44 (holotype CGMCC3.20145). GenBank: ITS = KX632531; TEF = KX632588; RPB2 = KX632645; ACL1 = KX632702; GPD = KX632759.

**Etymology:** *zoigense* (Latin), the specific epithet about the place where the type was found.

**Description:** Cultures and anamorph: optimal growth at 25 °C on all four media. On CMD after 72 h, growth is 25–28 mm at 20 °C and 28–31 mm at 25 °C. Colony is dense and has a wavy to crenate margin. Surface becomes distinctly zonate and white to grayish-green but celadon to atrovirens later, and it is granular in the center and distinctly radially downy outside and shows whitish surface hyphae and reverse-diffusing croci to pale brown pigment (Fig. 4a). Aerial hyphae are numerous to punctate and long, forming radial strands, with white mycelial patches appearing in aged cultures (Fig. 4e). Autolytic excretions are rare, with no coilings observed. Conidiation was noted after 3–4 d at 25 °C, a yellow or greenish color appears after 7 days, conidiation is effuse, and in intense tufts, erect conidiophores occur around the plug and on aerial hyphae. They are mainly concentrated along the colony center, show a white color that turns green, and then finally degenerate, with conidia often adhering in chains. Conidiophores are short and simple with asymmetric branches. Branches produce phialides directly. Phialides are generally solitary along main axes and side branches and sometimes paired in the terminal position of the main axes, sometimes in whorls of 2–3. Phialides are 4.5–10.5 × 2–5 μm ($\overline{x }$ = 7.5 ± 1.5 × 3 ± 0.5, n = 50) and 1.5–2.5 μm ($\overline{x }$ = 2 ± 0.2) wide at the base, lageniform or ampulliform, mostly uncinate or slightly curved, less straight, and often distinctly widened in the middle (Fig. 4f–k). Conidia are 3–4.5 × 2.3–4 μm ($\overline{x }$ = 3.5 ± 0.3 × 3 ± 0.3, n = 50) and initially hyaline, and they turn green and are oblong or ellipsoidal, almost with constricted sides, and smooth, eguttulate or with minute guttules, with indistinct scars (Fig. 4m).

On PDA, after 72 h, growth is 35–41 mm at 20 °C and 50–55 mm at 25 °C; and mycelium covers the plate after 5 days at 25 °C. Colonies are dense with wavy to crenate margins; and mycelia are conspicuously differentiated in width of the primary and secondary hyphae. Surface becomes distinctly zonate, yellowish-green to prasinous in color and celadon to atrovirens later, and it is farinose to granular in the center, distinctly radially downy outside, with whitish of surface hyphae and reverse-diffusing brilliant yellow to fruit-green pigment (Fig. 4c). Aerial hyphae are numerous, long and ascend several millimeters, forming radial strands, with white mycelial patches appearing in aged cultures. Autolytic excretions are rare; and no coilings are observed. Odor is indistinct or fragrant. Chlamydospores examined after 7 days at 4.5–9 × 4.5–7.5 μm ($\overline{x }$ = 6 ± 1.1 × 6 ± 0.7, n = 50), and they are terminal, intercalary, globose or ellipsoidal, and smooth (Fig. 4l). Conidiation is noted after 3–4 days and yellow or greenish after 7 days. Conidiophores are short and simple with asymmetric branches; conidia are greenish, ellipsoidal, and smooth.

On SNA, after 72 h, growth is 13–15 mm at 20 °C and, 16–21 mm at 25 °C; and mycelium covers the plate after 12–13 days at 25 °C. Colony is similar to that on CMD, with a little wave margin, although mycelia are looser and slower on the agar surface. Aerial hyphae are relatively inconspicuous and long along the colony margin. Autolytic activity and coiling are absent or inconspicuous. No diffusing pigment or distinct odor are produced (Fig. 4d). Conidiation was noted after 3–4 days at 25 °C, and many amorphous, loose white or aqua cottony tufts occur, mostly median from the plug outwards, and they are confluent to masses up and white but then turn green. After 4–5 days, conidiation becomes dense within the tufts, which are loose at their white margins with long, straight, or slightly sinuous sterile ends in the periphery. Tufts consisting of a loose reticulum with branches often at right angles, give rise to several main axes. Main axes are regular and tree-like, with few or many paired or unpaired side branches. Branches are flexuous, and phialides are solitary along the main axes and side branches, and they are sometimes paired in the terminal position of the main axes, sometimes in whorls of 2–3 that are often cruciform or in pseudo-whorls up to 4. Phialides and conidia are similar to that on CMD.

### New records for China

**Figure 5:**
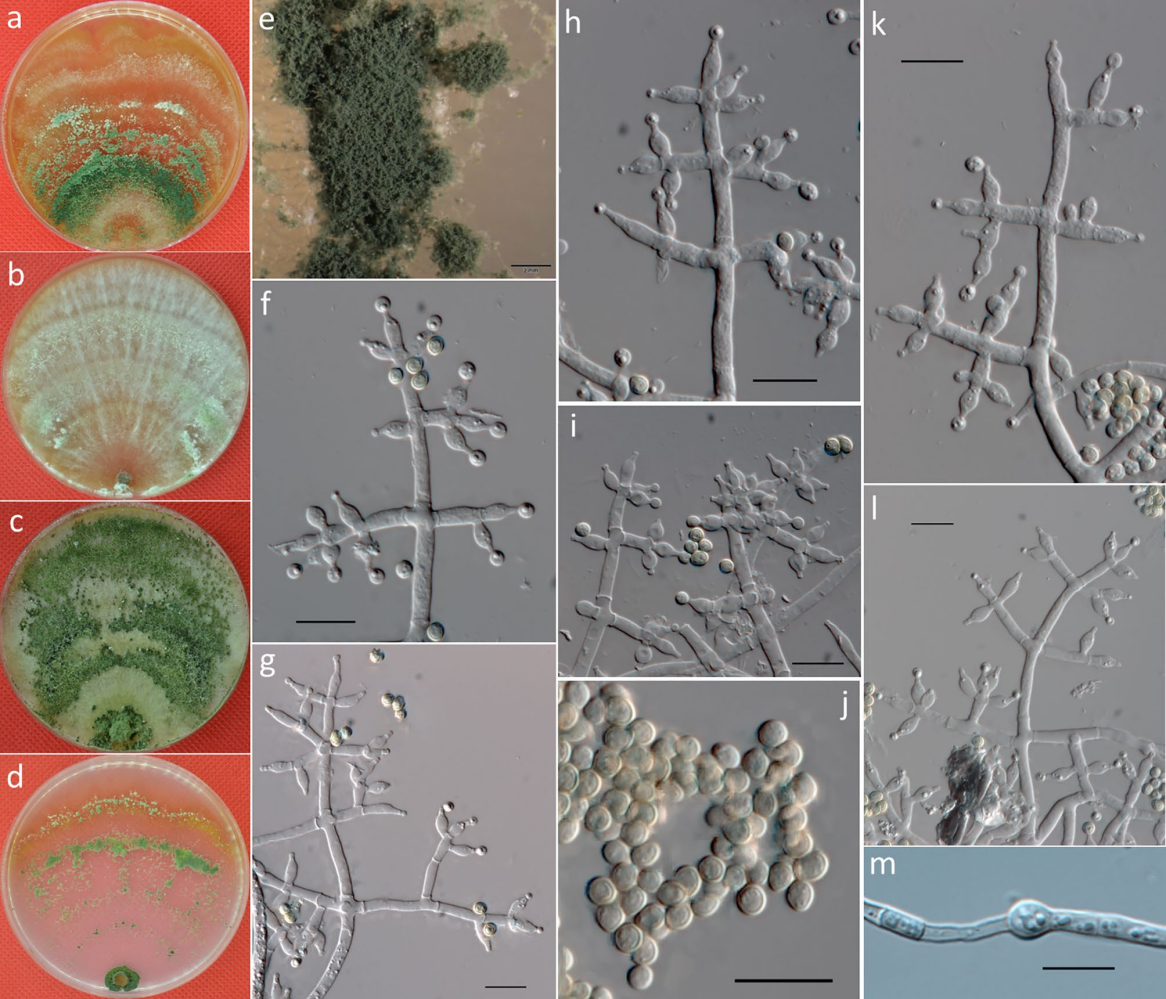
Cultures and asexual morph of *Trichoderma atrobrunneum*. (**a–d**) Cultures at 25 °C [(**a**) on CMD, 7 days; (**b**) on MEA, 4 days; (**c**) on PDA, 15 days; and (**d**) on SNA, 7 days]. (**e**) Conidiation tuft (SNA, 7 days). (**f–i**,**k**,**l**) Conidiophores and phialides (CMD, 5–7 days). (**j**) Conidia (CMD, 6 days). (**m**) Chlamydospores (PDA, 7 days). Scale bars: (**e**) = 2 mm; (**f–m**) = 10 μm.

***Trichoderma atrobrunneum*** F. B. Rocha et al., Mycologia 107: 571, 2015 (Fig. 5).

**Specimen examined:** CHINA. SICHUAN PROVINCE: Zoige Alpine Wetland, on soil, 29 June 2013, *G.S. Gong T42* (holotype CGMCC.20167). GenBank: ITS = KX632514; TEF = KX632571; RPB2 = KX632628; ACL1 = KX632685; GPD = KX632742.

**Description:** Cultures and anamorph: optimal growth at 25 °C on all media. On CMD, after 72 h, growth is 35–37 mm at 20 °C and 46–53 mm at 25 °C; mycelium covers the plate after 5–6 days at 25 °C. Colonies show distinct zonation. Mycelia are loose and thin; hyphae are narrow, sinuous and often form strands on the margin (Fig. 5a). Aerial hyphae are slight, forming a thin white to green downy fluffy or floccose mat. The light brown or brown pigment is observed, with no distinct odor noted. Conidiophores are pyramidal, often with opposing and somewhat widely spaced branches, with the main axis and each branch terminating in a cruciate, sometimes verticillate, whorl of up to four phialides. Phialides are ampulliform to lageniform and 4.9–7.6 × 2.2–3.0 μm ($\overline{x }$ = 6 ± 0.7 × 2.5 ± 0.2, n = 50) and 1.5–2.5 μm ($\overline{x }$ = 1.5 ± 0.3) wide at the base (Fig. 5f–i,k,l). Conidia are 2.5–4 × 2.5–3.5 μm ($\overline{x }$ = 3 ± 0.3 × 3 ± 0.2, n = 50), yellow to green, smooth, and circular to ellipsoidal (Fig. 5j).

On PDA, after 72 h, growth is 41–43 mm at 20 °C and 50–55 mm at 25 °C; and mycelium covers the plate after 5–6 days at 25 °C. Colonies show indistinct zonation. Mycelia are dense, opaque, and thick; hyphae are wide, sinuous and often form strands on the margin (Fig. 5c). Margin is thick and defined. Aerial hyphae are abundant and form a thick green downy mat. Conidiation forms abundantly within 4 days in broad concentric rings. Chlamydospores examined after 7 days are 5–9 × 5.5–8.5 μm ($\overline{x }$ = 6.5 ± 0.9 × 6.5 ± 0.9, n = 30), globose when terminal, smooth, and intercalary (Fig. 5m).

On SNA, after 72 h, growth is 33–35 mm at 20 °C and 38–40 mm at 25 °C; and mycelium covers the plate after 7–8 days at 25 °C. Colonies show distinct zonation. Mycelia are thin and yellow to green; hyphae are wide and sinuous, with indistinct strands on the margin (Fig. 5d). Margin is thin and ill-defined. Aerial hyphae are slight, forming a thin green downy fluff appearing in the colony (Fig. 5e). Diffusing pigment was observed in a ring, and no distinct odor was noted. Conidiation is similar to CMD.

### Accepted species previously reported in China

**Figure 6:**
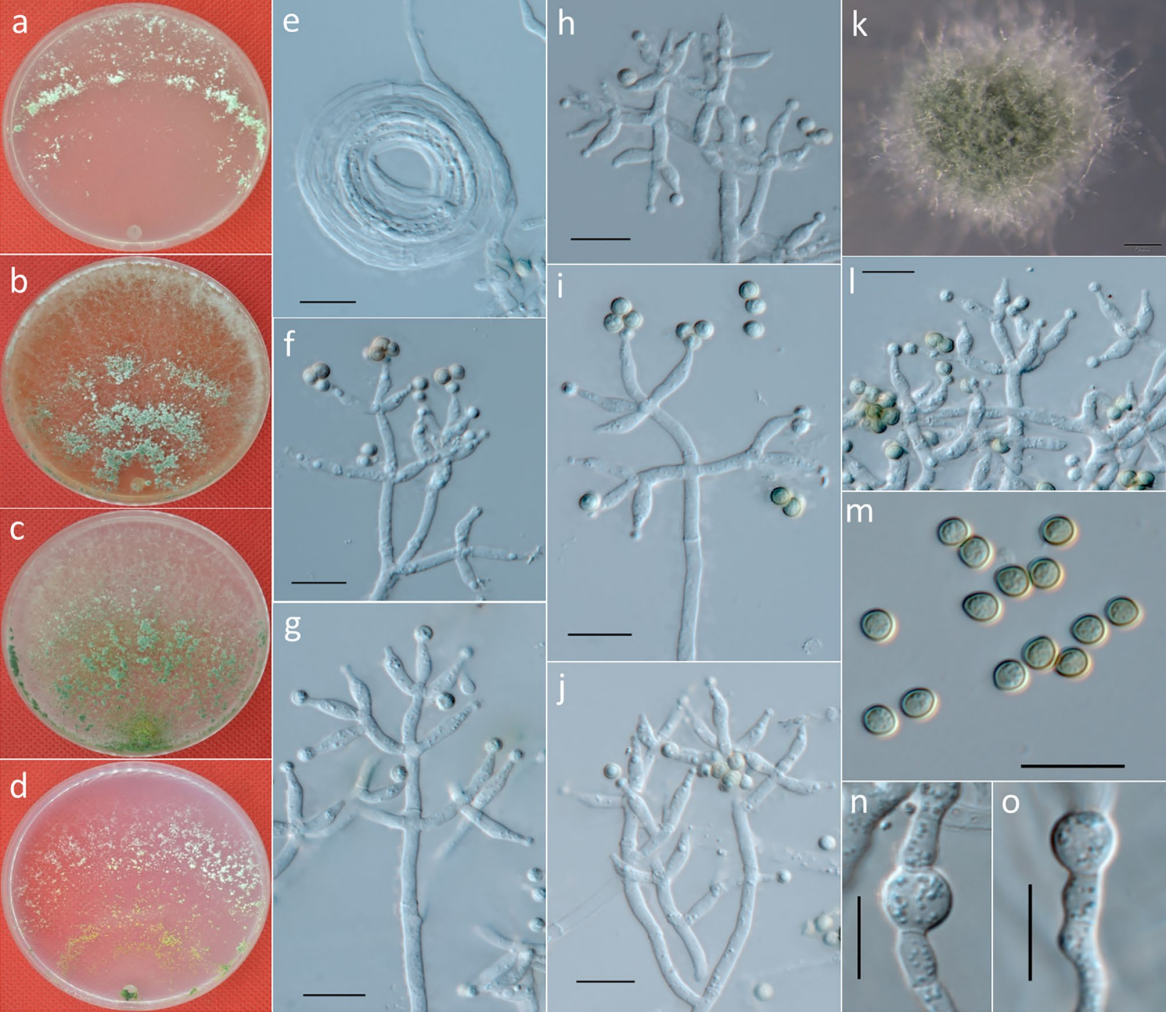
Cultures and asexual morph of *Trichoderma alni*. (**a**–**d**). Cultures after 7 days at 25 °C [(**a**) on CMD; (**b**) on MEA; (**c**) on PDA; and (**d**) on SNA]. € Coilings of aerial hyphae (PDA, 6 days). (**f**–**j**,**l**). Conidiophores and phialides (CMD, 5–7 days). (**k**) Conidiation tuft (PDA, 7 days). (**m**) Conidia (CMD, 6 days). (**n**,**o**) Chlamydospores (PDA, 7 days). Scale bars: (**e**–**j,l**–**o**) = 10 μm; (**k**) = 2 mm.

***Trichoderma alni*** Jaklitsch, Mycologia 100: 799. 2008 (Fig. 6).

***Description*:** Cultures and anamorph: Optimum growth at 25 °C on all media; no growth at 35 °C. On CMD, after 72 h, growth of 34–36 mm at 20 °C and 50–51 mm at 25 °C; and mycelium covers the plate after 5–6 days at 25 °C. Colonies show distinct zonation. Mycelia are loose and thin; hyphae are narrow and sinuous and often form strands on the margin (Fig. 6a). Aerial hyphae are slight and form a thin white to green downy, fluffy or floccose mat. No diffusing pigment or distinct odor is noted. Conidiophores are hyaline and thick, with side branches on several levels at the base of the elongations that are mostly paired and in right angles with phialides in whorls of 3–5. Phialides are 5.5–11.5 × 2–3.5 μm ($\overline{x }$ = 8 ± 1.4 × 2.5 ± 0.4, n = 50) and 1.5–2.5 μm ($\overline{x }$ = 2 ± 0.4) wide at the base, often short and wide, and ampulliform (Fig. 6f–j,l). Conidia are 3–4 × 2.5–3.5 μm ($\overline{x }$ = 3.5 ± 0.2 × 3 ± 0.2, n = 50), dark green, smooth, and ellipsoidal (Fig. 6m).

On PDA, after 72 h, growth is 33–35 mm at 20 °C and 41–43 mm at 25 °C; and mycelium covers the plate after 6–7 days at 25 °C. Colonies show indistinct zonation. Mycelia are dense, opaque, and thick; hyphae are wide, sinuous and often form strands on the margin (Fig. 6c). Margin is thin and ill defined. Aerial hyphae are slight, coiled (Fig. 6e), forming a thin white to green downy, fluffy or floccose mat (Fig. 6k). Chlamydospores examined after 7 days are 6–9.5 × 5–8 μm ($\overline{x }$ = 7.5 ± 0.9 × 7 ± 0.9, n = 30), globose to oval when terminal, and smooth, and few are intercalary (Fig. 6n,o).

On SNA, after 72 h, growth is 18–19 mm at 20 °C and 28–32 mm at 25 °C; and mycelium covers the plate after 6–7 days at 25 °C. Colonies show distinct zonation. Mycelia are thin and yellow to green; hyphae are wide and sinuous and show indistinct strands on the margin (Fig. 6d). Margin is thin and ill-defined. Aerial hyphae are slight and form a thin white downy, fluffy, or floccose mat appearing in distal parts of the colony. No diffusing pigment or distinct odor was noted. Conidiation is similar to CMD.

**Figure 7:**
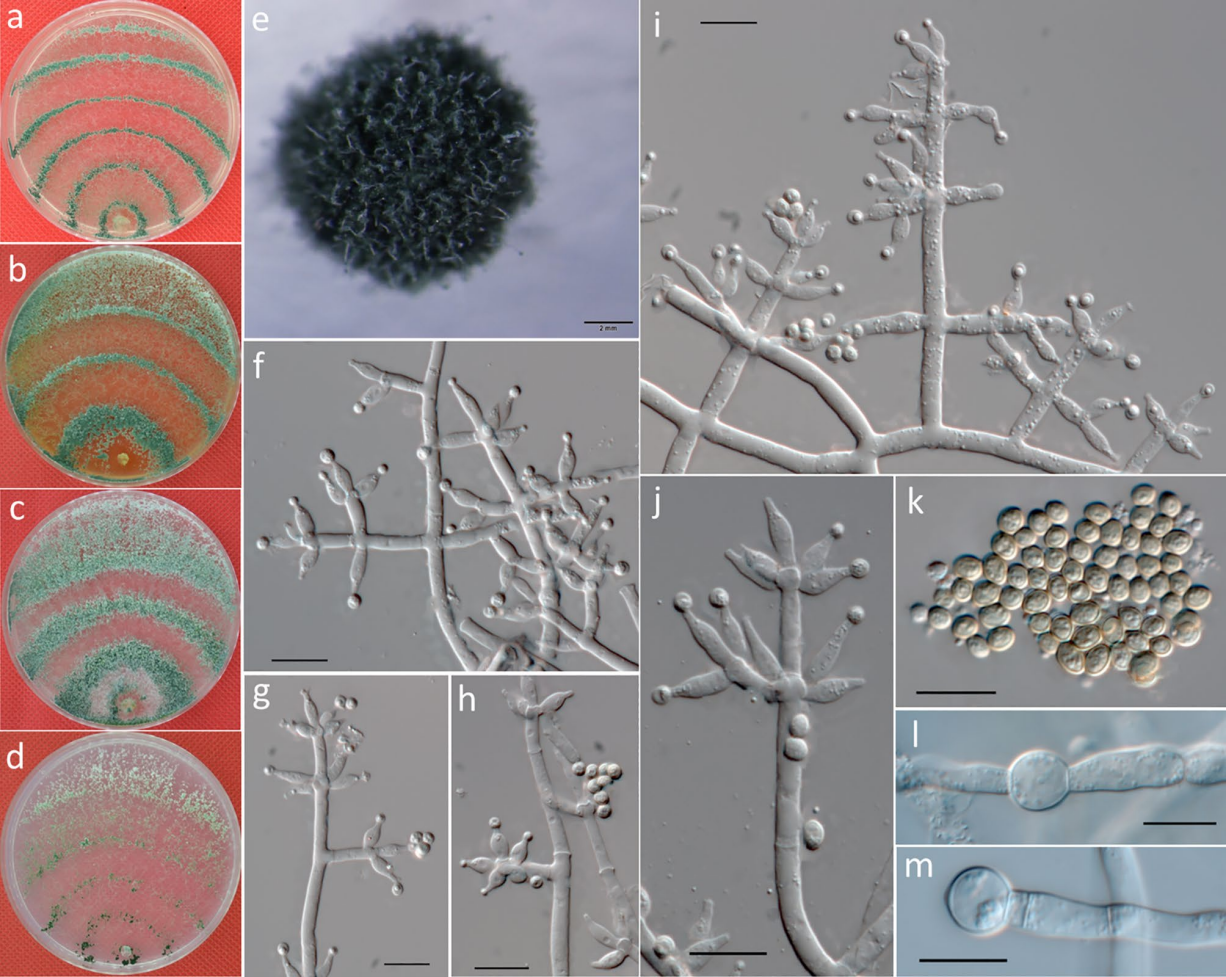
Cultures and asexual morph of *Trichoderma harzianum*. (**a–d**) Cultures after 7 days at 20 °C [(**a**) on CMD; (**b**) on MEA; (**c**) on PDA; and (**d**) on SNA]. (**e**) Conidiation tuft (CMD, 7 days). (**f–j**) Conidiophores and phialides (CMD, 5–7 days). (**k**) Conidia (CMD, 5 days). (**l**,**m**) Chlamydospores (PDA, 7 days). Scale bars: (**e**) = 2 mm; (**f–m**) = 10 μm.

***Trichoderma harzianum*** Rifai, Mycol. Pap. 116: 38, 1969 (Fig. 7).

***Description*:** Cultures and anamorph: optimal growth at 25 °C on all media. On CMD, after 72 h, growth is 34–38 mm at 20 °C and 46–53 mm at 25 °C; mycelium covers the plate after 5–6 days at 25 °C. Colonies show distinct zonation. Mycelia are loose and thin; hyphae are narrow, sinuous, and often form strands on the margin (Fig. 7a). Aerial hyphae are abundant and radiating and form thick green downy, fluffy, or floccose mats (Fig. 7e). No diffusing pigment, but fragrant odor noted. Conidiophores are pyramidal with opposing branches, with each branch terminating in a cruciate whorl of up to four or five phialides. Phialides are frequently solitary or in a whorl of three or four. Phialides are ampulliform to lageniform and often constricted below the tip to form a narrow neck of 4.5–8 × 2–3.5 μm ($\overline{x }$ = 6 ± 0.8 × 2.5 ± 0.3, n = 50) and 1–2.5 μm ($\overline{x }$ = 2 ± 0.3) wide at the base (Fig. 7f–j). Conidia are subglobose to ovoid, 3–4.5 × 2.5–3.3 μm ($\overline{x }$ = 3.5 ± 0.3 × 3 ± 0.2, n = 50), laurel-green to bright green, smooth, and ellipsoidal (Fig. 7k).

On PDA, after 72 h, growth is 41–43 mm at 20 °C and 50–55 mm at 25 °C; and mycelium covers the plate after 5–6 days at 25 °C. Colonies show distinct zonation. Mycelia are dense, opaque, and thick; hyphae are wide and sinuous and often form strands on the margin (Fig. 7c). Margin is thick and ill defined. Aerial hyphae are abundant and radiating and form thick green downy, fluffy or floccose mats. Chlamydospores examined after 7 days are 5.5–9 × 5.5–9.0 μm ($\overline{\mathrm{x} }$ = 7 ± 0.8 × 7 ± 0.8, n = 30), globose to oval when terminal and smooth, showing an almost unobserved intercalary (Fig. 7l,m).

On SNA, after 72 h, growth is 33–35 mm at 20 °C and 38–40 mm at 25 °C; and mycelium covers the plate after 7–8 days at 25 °C. Colonies show distinct zonation. Mycelia are thin and green; hyphae are narrow and sinuous and show indistinct strands on the margin (Fig. 7d). Margin is thin and ill defined. Aerial hyphae are slight and form a thick downy, fluffy, or floccose mat appearing in the colony. No diffusing pigment or distinct fragrant odor was noted. Conidiation was similar to CMD.

**Figure 8:**
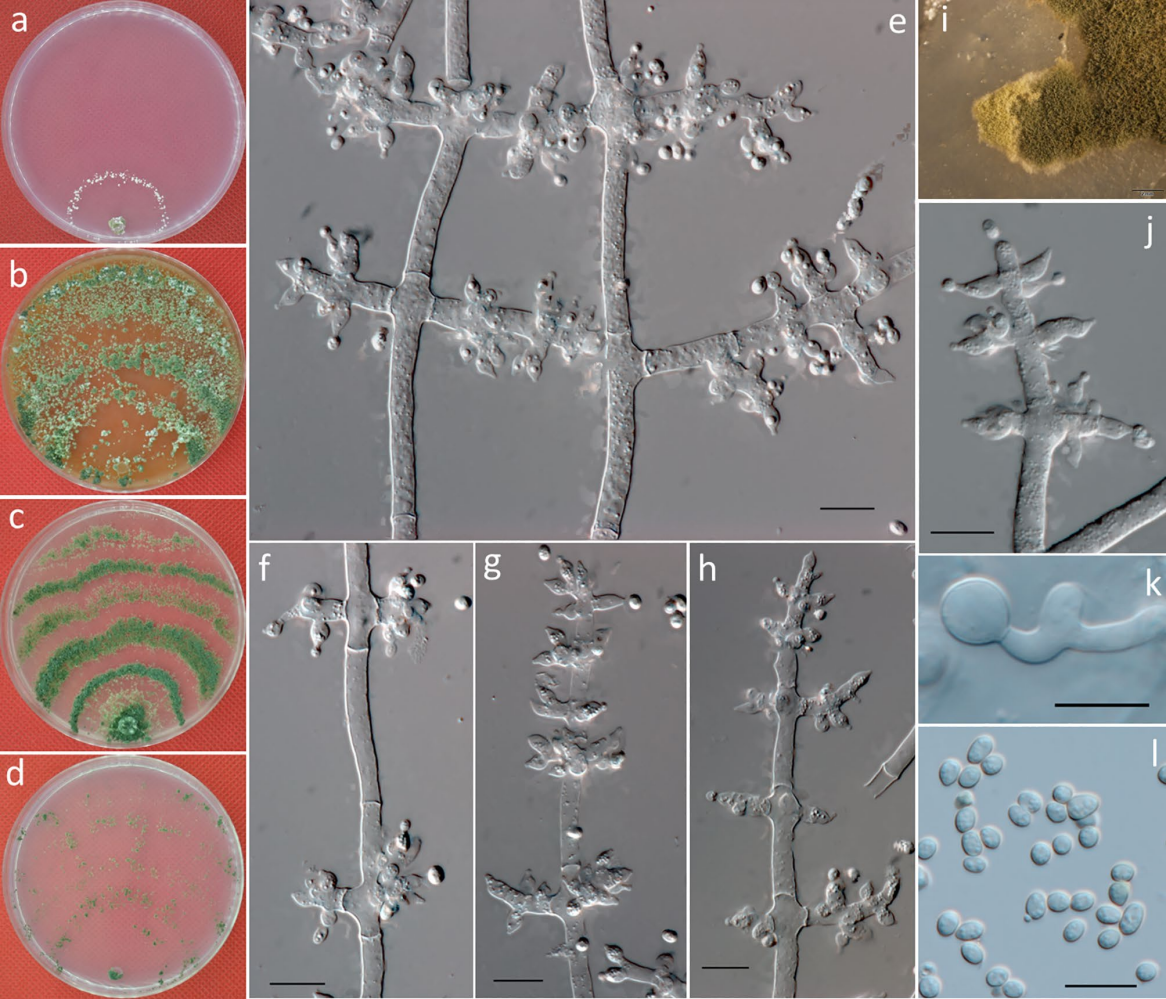
Cultures and asexual morph of *Trichoderma polysporum*. (**a–d**) Cultures at 20 °C [(**a**) on CMD, 7 days; (**b**) on MEA, 15 days; (**c**) on PDA, 15 days; and (**d**) on SNA, 15 days]. (**i**) Conidiation tuft (PDA, 15 days). (**e**–**h,j**) Conidiophores and phialides (CMD, 5–7 days). (**k**) Chlamydospores (CMD, 7 days). (**l**) Conidia (PDA, 6 days). Scale bars: (**i**) = 2 mm; (**e–h,j**) = 10 μm.

***Trichoderma polysporum*** Rifai, Mycol. Pap. 116: 18, 1969 (Fig. 8).

***Description*:** Cultures and anamorph: optimal growth at 20 °C on all media, no growth at 35 °C. On CMD, after 72 h, growth is 14–16 mm at 20 °C and 9–12 mm at 25 °C; and mycelium covers the plate after 9–10 days at 20 °C. A colony is hyaline, thin and loose, with little mycelium on the agar surface, and it is indistinctly zonate but becomes zonate by conidiation in white tufts after 4–5 d and grass green to green after 6 days (Fig. 8a). Aerial hyphae are long and dense and forming little greenish aggregates that are granular to pulvinate. No pigment or odor. Conidiation noted after 4–5 days, and it is white to greenish, with sterile smooth to rough helical elongations in the distal zones from pustules. Conidiophores are hyaline and thick with side branches on several levels at the base of the elongations that are mostly paired and at right angles with phialides in whorls of 2–5. Phialides are 5–10.5 × 2.5–4 μm ($\overline{x }$ = 7 ± 1.9 × 3.5 ± 0.4, n = 50) and 2–4 μm ($\overline{x }$ = 3 ± 0.5) wide at the base, often short and wide and ampulliform (Fig. 8e–h,j). Conidia are 2.5–4 × 2–3 μm ($\overline{x }$ = 3.5 ± 0.4 × 2.5 ± 0.2, n = 50), hyaline, smooth, and ellipsoidal (Fig. 10l).

On PDA, after 72 h, growth is 24–26 mm at 20 °C and 13–16 mm at 25 °C; and mycelium covers the plate after 8–9 days at 20 °C. A colony is densest, distinctly zonate, and grass green to spearmint green; mycelia are conspicuously dense; and surface hyphae form radial strands (Fig. 8c). Aerial hyphae are long and dense and form greenish aggregates that are granular to pulvinate (Fig. 8i). No diffusing pigment and odor. Chlamydospores examined after 7 days are 5.5–9 × 5–7.5 μm ($\overline{x }$ = 7 ± 0.9 × 6 ± 0.6, n = 30), globose to oval when terminal, and smooth, with an almost unobserved intercalary (Fig. 8k).

On SNA, growth is approximately 7 mm/day at 20 °C and 5 mm/day at 25 °C; and mycelium covers the plate after 10 days at 20 °C. A colony is hyaline, thin, and loose, with little mycelium on the agar surface, not or indistinctly zonate, but becomes zonate by conidiation in white tufts after 4–5 days; and the margin is downy by long aerial hyphae, which degenerating/dissolving soon (Fig. 8d).

**Figure 9:**
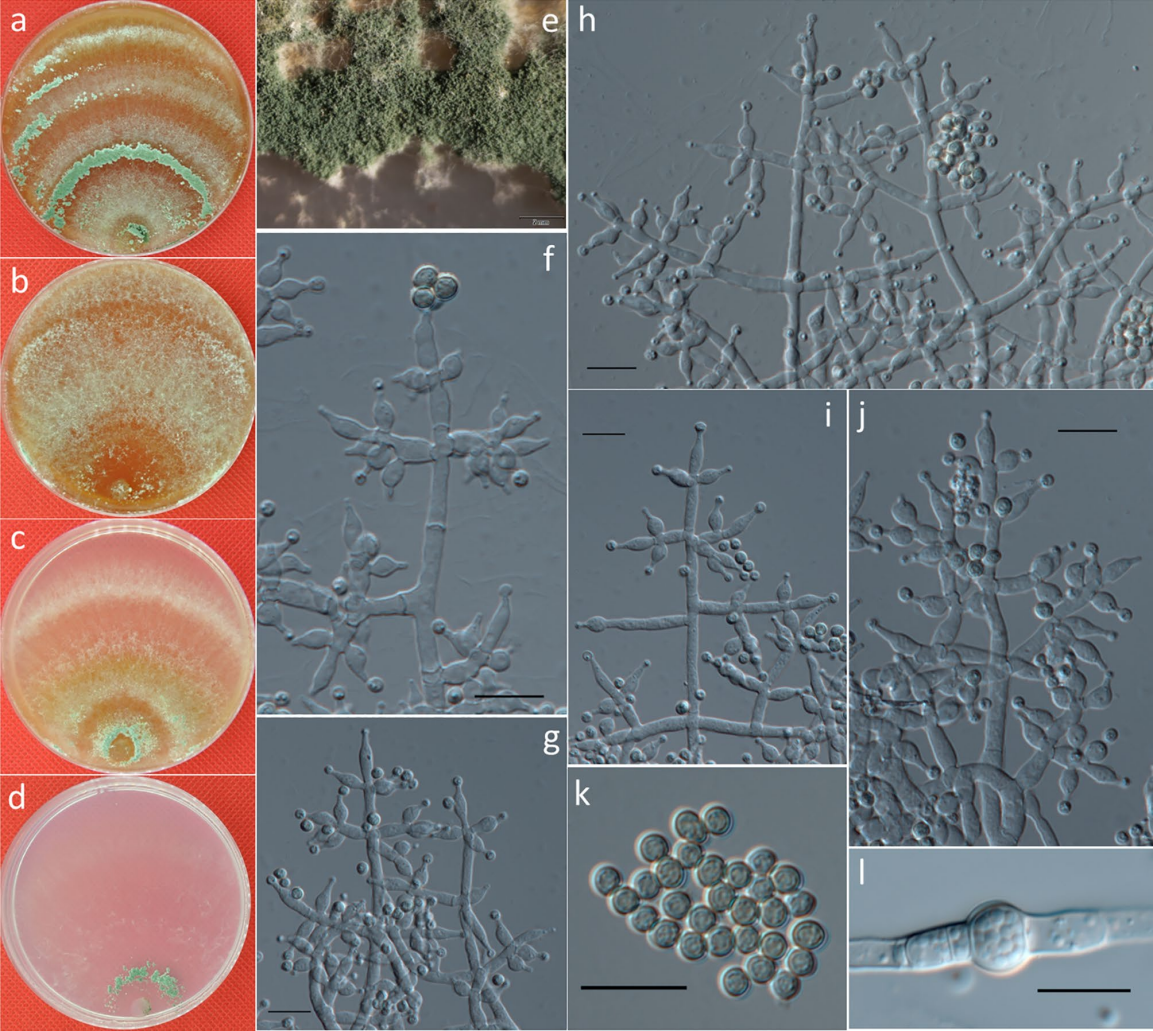
Cultures and asexual morph of *Trichoderma pyramidale*. (**a–d**) Cultures at 25 °C [(**a**) on CMD, 7 days; (**b**) on MEA, 4 days; (**c**) on PDA, 4 days; and (**d**) on SNA, 4 days]. (**e**) Conidiation tuft (PDA, 7 days). (**f**–**j**) Conidiophores and phialides (CMD, 5–7 days). (**k**) Conidia (CMD, 6 days). (**l**) Chlamydospores (PDA, 7 days). Scale bars: (**e**) = 2 mm; (**f–l**) = 10 μm.

***Trichoderma pyramidale*** W. Jaklitsch & P. Chaverri, Mycologia 107: 581, 2015 (Fig. 9).

***Description*:** Cultures and anamorph: optimal growth at 25 °C on all media, with little growth at 35 °C. On CMD, after 72 h, growth is 29–32 mm at 20 °C and 48–53 mm at 25 °C; and mycelium covers the plate after 5–6 days at 25 °C. Colonies show distinct zonation. Mycelium is loose and thin; hyphae are narrow, sinuous, and often form strands on the margin (Fig. 9a). Aerial hyphae are slight, forming a thin white to green downy, fluffy or floccose mat. Brown pigment is shown, but no distinct odor noted. Conidiophores are hyaline and thick with side branches on several levels at the base of the elongations that are mostly paired and at right angles with phialides in whorls of 3–5. Phialides are 5–9.5 × 2.5–3 μm ($\overline{x }$ = 7 ± 1.1 × 3 ± 0.3, n = 50) and 1–2.5 μm ($\overline{x }$ = 1.5 ± 0.3) wide at the base and often short, wide, and ampulliform (Fig. 9f–j). Conidia are 2.5–4 × 2.5–3.5 μm ($\overline{x }$ = 3.5 ± 0.3 × 3 ± 0.2, n = 50), green, smooth, and ellipsoidal (Fig. 9k).

On PDA, after 72 h, growth is 41–43 mm at 20 °C and 50–55 mm at 25 °C; and mycelium covers the plate after 5–6 days at 25 °C. Colonies show indistinct zonation. Mycelia are dense, opaque, and thick; hyphae are wide, sinuous and often form strands on the margin (Fig. 9c). Margin is thin and ill defined. Aerial hyphae are slight and form a thin white to green downy, fluffy or floccose mat (Fig. 9e). Chlamydospores examined after 7 days are 5.5–10 × 5.5–10 μm ($\overline{x }$ = 7 ± 0.9 × 7 ± 0.9, n = 30), globose to oval when terminal or intercalary, and smooth (Fig. 9l).

On SNA, after 72 h, growth is 33–35 mm at 20 °C and 38–40 mm at 25 °C; and mycelium covers the plate after 7–8 days at 25 °C. Colonies show distinct zonation. Mycelium is thin, yellow to green; hyphae are wide, sinuous, with indistinct strands on the margin (Fig. 9d). Margin is thin and ill defined. Aerial hyphae are slight and form a thin white downy, fluffy or floccose mat in distal parts of the colony. No diffusing pigment or distinct odor noted. Conidiation similar to CMD.

**Figure 10:**
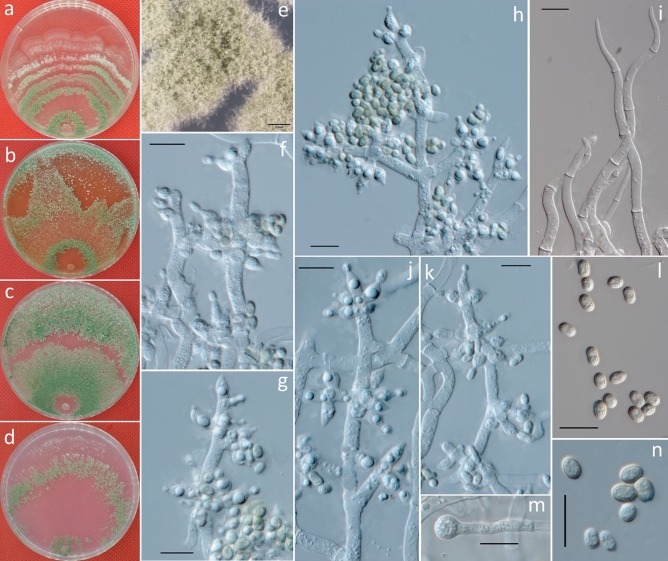
Cultures and asexual morph of *Trichoderma rossicum*. (**a–d**) Cultures after 7 days at 25 °C [(**a**) on CMD; (**b**) on MEA; (**c**) on PDA; and (**d**) on SNA]. € Conidiation tuft (PDA, 7 days). (**f–h,j,k**) Conidiophores and phialides (CMD, 5–7 days). (**i**) Elongations (CMD, 6 days). (**l,n**) Conidia (CMD, 6 days). (**m**) Chlamydospores (PDA, 7 days). Scale bars: (**e**) = 2 mm; (**f–n**) = 10 μm.

***Trichoderma rossicum*** Bissett et al*.*, Canad. J. Bot. 81: 578, 2003 (Fig. 10).

***Description*:** Cultures and anamorph: optimal growth at 25 °C on all media. On CMD, growth of 10–11 mm/day at 20 °C and 15–17 mm/day at 25 °C; and mycelium covers the plate after 6–7 days at 20 °C. Colony is dense with a wavy margin, and the surface becomes distinctly zonate (Fig. 10a). Aerial hyphae are numerous, long, elongate, and villiform in the plate (Fig. 10i). No diffusing pigment or odor. Autolytic activity is variable, and coilings are scarce or inconspicuous. Conidiation noted after 3–4 days at 20 °C. Conidiation is effuse and in intense tufts that are hemispherical or irregular, and they show wide wheel grain banding that is gray green to deep green. Conidiophores radiate from the reticulum and are broad, straight, sinuous or helically twisted, show distally slightly pointed elongations, taper from the main axes to top branches, and present primary branches arranged in pairs or in whorls of 2–3, with secondary branches to solitary. Phialides are 4.5–14 × 2.5–4 μm ($\overline{x }$ = 7 ± 1.5 × 3.5 ± 0.3, n = 50) and 2–3.5 μm ($\overline{x }$ = 3 ± 0.4) wide at the base, ampulliform, and in whorls of 3–6 (Fig. 10f–h,j,k). Conidia are 3.5–5.5 × 2.5–4 μm ($\overline{x }$ = 4.5 ± 0.5 × 3 ± 0.2, n = 50), short cylindrical, and a gray color when single and pea green to yellow green in a group (Fig. 10l,n).

On PDA, growth is 12–15 mm/day at 20 °C, 12–16 mm/day at 25 °C; and mycelium covers the plate after 4–5 days at 25 °C. Colony is denser with a wavy margin than that on CMD, and the surface is distinctly zonate (Fig. 10c). Aerial hyphae are numerous, long, and villiform to pulvinate in the plate. No diffusing pigment and odor (Fig. 10e). Autolytic activity is variable, coilings are scarce or inconspicuous. Chlamydospores examined after 7 days are 6.5–9.5 × 6–9 μm ($\overline{x }$ = 7 ± 1.0 × 7 ± 0.9, n = 30), terminal and intercalary, globose or ellipsoidal, and smooth (Fig. 10m).

On SNA, growth is 8–13 mm/day at 20 °C and 8–12 mm/day at 25 °C; and mycelium covers the plate after 6–7 day at 25 °C. Colony is hyaline, thin and dense; and mycelium degenerate rapidly (Fig. 10d). Aerial hyphae are inconspicuous, autolytic activity is scant, and coilings are distinct. Conidiation noted after approximately 4 days and starts in white fluffy tufts spreading from the center to form concentric zones, and they compact to pustules with a white to greenish color.

## Discussion

To characterize the biodiversity and establish the species composition of *Trichoderma* associated with soil in the Zoige alpine wetland ecological region of Southwest China, morphological characteristics and multilocus phylogenetic analyses were performed to identify 80 strains as *T. harzianum* (48 strains, 60%), *T. alni* (15 strains, 18.75%), *T. zoigense* (a new species, 8 strains, 10%), *T. rossicum* (4 strains, 5%), *T. atrobrunneum* (3 strains, 3.75%), *T. polysporum* (1 strain, 1.25%) and *T. pyramidale* (1 strain, 1.25%)*.* This is the first comprehensive report on the population structure of *Trichoderma* in the Zoige alpine wetland. A specialized analysis of *Trichoderma* from 100 soil samples shows a high richness of the *Trichoderma* species in this region and indicates the presence of latent resources, due to their complex natural environment and unique climatic conditions. Zoige alpline wetland is generally considered the most important carbon sink ecosystem, in which soil microflora and fungi play vital roles in biogeochemical cycles. So, we should focus on *Trichoderma* species that contributes to carbon (nutrient) cycles and other functions in Zoige alpine wetlands in subsequent studies.

In this study, the high throughput amplicon sequencing (HTAS) approach based on ITS have used to evaluate species diversity of *Trichoderma* spp., but showed ineffectively. 13 OTUs of *Trichoderma* spp. were obtained from 11 soil samples. However, because the single ITS region is not accurate for determining species of *Trichoderma*, the diversity of the genus remains unclear based on the high-throughput sequencing results. Therefore, the data have not been shown in the study.

Although many studies have focused on identifying *Trichoderma,* identifying *Trichoderma* species based on only morphological characteristics remains difficult. Amplifying four universal fungal genes, *gpd*, *acl1*, *rpb2* and *tef1*, showed that the *gpd* gene could divide approximately the 57 representative strains into 4 clades, which were precisely aligned with the previous 4 morphological groups. The *gpd* gene was suitable for categorizing large groups but was not helpful for the accurate identification of speciation within the *Trichoderma* complex^71^. In fact, any single gene among *acl1*, *rpb2* and *tef1* can play an essential role in identifying *Trichoderma* species but cannot accurately distinguish *Trichoderma* at the species level. Notably, although the primer pair EF1-728F and TEF1LLErev for *tef1* was helpful, it did not always successfully amplify all tested DNA materials. Admittedly, many factors affect PCR amplification, not all of which can be attributed to primers, among which the quality of DNA may also be one of the factors. Phylogenetic studies of many species have proven that the most accurate method of species identification is to combine phylogenetic analysis with morphological phenotypic characteristics. In this study, when the genes *acl1*, *rpb2* and *tef1* were used in multilocus phylogenetic analysis, the phylogenetic relationships among taxa were consistent with those identified in previous studies in which the phylogenetic tree was built based on the genes *rpb2* and *tef1* either singly or in combination^46,47,49,56^.

We found that the Longibrachiatum clade contained a new species, *T. zoigense*, which was phylogenetically distinct from any other species of *Trichoderma* (Fig. 2) and provided a low level of support for relationships with *T. citrinoviride* (C.P.K. 2005) and *T. saturnisporum* (ATCC 18,903) (Fig. 2, MPBP = 62%). Compared to their morphological characteristics of the above two species, *T. zoigense* was challenging to distinguish from *T. citrinoviride* and *T. saturnisporum* by colony and spores. However, *T. zoigense* produced yellow pigment dispersion and a fragrance in all tested media and easily produced chlamydospores^42,46,47,52,71^.

The results of our studies demonstrated significant differences in the abundance and distribution of *Trichoderma* species isolated in the Zoige alpine wetland natural region. *T. harzianum* showed the highest abundance among the species isolated from five soil layers and four soil types, implying that this species had good adaptability and could survive under most environmental conditions. Only *T. polysporum* was isolated at a soil depth of 50–100 cm, indicating that it prefers to live in a low-temperature environment^72^. In general, it is assumed that some *Trichoderma* species have stricter requirements for the growth environment and, thus, a narrower range for survival^73^.

## Conclusion

In conclusion, seven *Trichoderma* species were identified from 100 soil samples collected from Zoige alpine wetland ecological regions, and *T. harzianum* was the preponderant species. The recognition of *Trichoderma zoigense* was described for the first time as a new species, and *T. atrobrunneum* as a new record for China was found. The results of our research will provide a reference for a greater understanding of soil microorganisms, ecological rehabilitation and reconstruction, and as microbial resources in the Zoige alpine wetland.

## Materials and methods

### Study region

The Zoige alpine wetland (32° 10′ ~ 34° 10′ N, 101° 45′ ~ 103° 55′ E) is located in the northwest part of Sichuan Province in China and on the eastern edge of the Qinghai-Tibet Plateau and has an average altitude of 3400 m above sea level and an area of 19,600 km^2^. It is a relatively pristine natural area with an annual temperature of 0.6–1.0 °C and an annual precipitation level of 580–860 mm. The cold, humid weather slows the decomposition of the soil organic matter and facilitates its accumulation in the soil^74–76^. Peat soil, meadow soil, subalpine meadow soil and aeolian sandy soil are extensively developed and the most common soil types in this area, because of its unique ecological conditions.

### Isolates and specimens

A total of 100 soil samples were collected across a range of soil types (peat soil, meadow soil, subalpine meadow soil and aeolian sandy soil) and soil layers (depth 0–10, 10–20, 20–30, 30–50, and 50–100 cm) in the Zoige alpine wetland ecological regions. Global positioning system technology (GPS Map 76; Garmin Ltd, USA) was used to determine the sampling locations. After removal of vegetation debris, approximately 300 g of each soil sample was immediately placed in a sterile plastic bag in a cooler, transported to the laboratory within 48 h and then stored at 4 °C.

Soil fungi were isolated using the suspension plating method^77^. Briefly, suspensions (1 mL) of various dilutions (10^–1^, 10^–2^ and 10^–3^) were placed on 90 mm diameter petri plates and Martin medium was then added and mixed evenly with the suspension. The plates were kept in the dark at 25 °C for 5 days, and the colonies of fungi were observed and counted. Three replicates were performed for each concentration. According to the colony characteristics, the purified fungal colonies were transferred onto potato dextrose agar (PDA) and kept in tube slants and glycerol for further taxonomic identification. The specimens were deposited in the Fungal Herbarium of Sichuan Agricultural University, with accession numbers of T1–T80. Moreover, the holotype of new species and new record species were deposited in China General Microbiological Culture Collection Center (CGMCC), with accession numbers CGMCC3.20145 and CGMCC3.20167.

### Morphology and growth rate

Cultures were prepared and maintained as described previously^46,78^. Cultures used for the study of asexual morph micromorphology were grown on PDA, on CMD (cornmeal agar supplemented with 2% (w/v) D (+)-glucose-monohydrate) containing 0.02% (w/v) streptomycin sulfate (Solarbio, China) and 0.02% (w/v) neomycin sulfate (Solarbio), on SNA (low-nutrient agar)^68,79^ or occasionally on MEA (2% malt extract, 2% agar–agar) at 20 °C or 25 °C under a 12 h/12 h light/dark cycle with cool white fluorescent light during the light period.

Fungal colony characteristics were observed on the CMD, PDA, MEA and SNA media and grown under 12 h of white light and 12 h of darkness at 20 °C and 25 °C. Colony textures and the presence or absence of exudates were recorded using a stereomicroscope (OLYMPUS SZX16, Japan). Colony morphologies were observed weekly with a digital camera (Nikon D3100, Japan). Micromorphological characteristics were observed after 3–7 days or 14 days of cultivation, and microscopic observations were performed in 3% KOH. Chlamydospores were measured from 7 to 30-day-old cultures on CMD or SNA plates under a compound microscope using a 100 × objective. The following characteristics of each isolate were measured: length and width of conidia (n = 50), length of phialides (n = 50), width of phialides at the base (n = 50), and width of phialides at the widest point (n = 50). Nomarski differential interference contrast (DIC) was used for observations and measurements, and data were gathered using a Carl Zeiss microscope (Axio Imager Z2, Germany). Colors were determined with Methuen’s Handbook of Colour.

To identify the optimal growth temperature and differentiate growth rates of the species, 3 representative strains or all strains (≤ 3 in total) for each species were selected to determine the growth rate on CMD at five temperature levels (15 °C, 20 °C, 25 °C, 30 °C and 35 °C) as described previously with minor modifications^46^. The strains were pre-grown on PDA for 48 h or 72 h at 25 °C. For new cultures, 5-mm agar blocks were cut from the margin of the colonies and transferred to fresh medium from the edge of the 9-cm petri dish. The maximum colony radius was measured every day until the plates were entirely covered with mycelium. The growth rate was calculated by linear regression of t versus r (t = time of incubation and r = radius measured from the edge of the agar plug).

### Molecular characterization

DNA samples of representative isolates of 57 morphotypes, which were chosen according to the morphological and cultural characteristics, were extracted from pure cultures (72 h at 25 °C) for phylogenetic analysis as described by Barnes et al.^80^. Part of the nuclear rDNA ITS region was amplified by PCR using the primer pair ITS1 5′ TCCGTAGGTGAACCTGCGG3 and ITS4 5′ TCCTCCGCTTATTGATATGC^81^. A 1-kb fragment of RNA polymerase II subunit B (*rpb2*) was amplified using the primer pair fRPB2-5f 5′ GAYGAYMGWGATCAYTTYGG and fRPB2-7cr 5′ CCCATRGCTTGYTTRCCCAT^82^. A 1.2-kb fragment of translation elongation factor 1 alpha (*tef1*) was amplified using the primer pair EF1-728F 5′ CATCGAGAAGTTCGAGAAGG^83^ and TEF1LLErev 5′ AACTTGCAGGCAATGTGG^78^. A 0.9-kb fragment of the larger subunit of ATP citrate lyase (*acl1*) was amplified using the primers acl1-230up 5′ AGCCCGATCAGCTCATCAAG and acl1-1220low 5′ CCTGGCAGCAAGATCVAGGAAGT^84^. A 0.4-kb fragment of a partial sequence of the glyceraldehyde-3-phosphate dehydrogenase (*gpd*) gene region was amplified using the primers GDF1 5′ GCCGTCAACGACCCCTTCATTGA and GDR1 5′ GGGTGGAGTCGTACTTGAGCATGT^85,86^. The PCR mixtures (30 μL) contained 1 μL of genomic DNA (approximately 100 ng), 1 μL of each primer (10 mM), 12 μL of sterile deionized water, and 15 μL of 2 × PCR MasterMix (TIANGEN Co., China). Amplifications were performed in an Eppendorf PCR amplifier (Mastercycler nexus X2, Germany). PCR products were sequenced with an ABI 3730xl DNA Analyzer by Sangon Biotech (Shanghai, China).

### Phylogenetic analyses

For approximate identification, all sequences of the 57 strains listed in Table 2 were compared with the NCBI sequence database using the BLAST algorithm. The two markers (ITS and *gpd*) sequenced in the present study were analyzed separately.ClustalX^87^ aligned their closest matches, and a distance tree was built with the neighbor-joining (NJ) algorithm in MEGA v. 6.0 with 1000 bootstrap replicates^81,88^. Combined *rpb2*, *tef1* and *acl1* gene sequences were analyzed based on a multilocus dataset. Finally, a phylogenetic analysis was performed for the sequences of a total of 101 strains obtained from the present study or other references in previous studies and complemented with GenBank sequences^46,47^.

Maximum parsimony (MP) analyses of the combined DNA matrix was performed with PAUP* v. 4.0 b10^89^ using 1000 replicates of a heuristic search with the random addition of sequences. All molecular characteristics were unordered and given equal weight, and all gaps were treated as missing data. The stability of clades was evaluated by bootstrap analysis with 1000 replicates. Descriptive tree statistics for parsimony (tree length [TL], consistency index [CI], retention index [RI], related consistency [RC] and homoplasy index [HI]) were calculated.

### Relationship with ecological factors

The isolation frequency was calculated at the species level using the following formula:

$$\mathrm{F }= \frac{\mathrm{n}}{\mathrm{N}}\times 100\mathrm{\%},$$

where F is the isolation frequency (%), n is the number of species isolated from soil samples, and N is the number of total soil samples. The relationships between the isolation frequency, soil types, and soil layers were subsequently analyzed.

## Supplementary Information

Supplementary Information 1.

Supplementary Information 2.

## Data Availability

All DNA sequences generated in this study have been registered to GenBank(https://www.ncbi.nlm.nih.gov). Supplementary material contains GenBank accessions of the sequences generated at STable1, and other raw data. The holotype of new species and new record species were deposited in China General Microbiological Culture Collection Center (CGMCC), with accession numbers CGMCC3.20145 and CGMCC3.20167.

## References

[1] Mueller GM, Bills GF, Foster MS (2004). Biodiversity of Fungi: Inventory and Monitoring Methods.

[2] Gadd GM (2007). Geomycology: Biogeochemical transformations of rocks, minerals, metals and radionuclides by fungi, bioweathering and bioremediation. Mycol. Res..

[3] Hollister EB, Schadt CW, Palumbo AV, Ansley RJ, Boutton TW (2010). Structural and functional diversity of soil bacterial and fungal communities following woody plant encroachment in the southern Great Plains. Soil. Biol. Biochem..

[4] James AW, Brain CS, Neil JM, Alan CG (2012). Species and organ specificity of fungal endophytes in herbaceous grassland plants. J. Ecol..

[5] Tedersoo L, Bahram M, Toots M, Diedhiou AG, Henkel TW, Kjoller R, Morris MH, Nara K (2012). Towards global patterns in the diversity and community structure of ectomycorrhizal fungi. Mol. Ecol..

[6] Wardle DA, Yeates GW, Barker GM, Bonner KI (2006). The influence of plant litter diversity on decomposer abundance and diversity. Soil. Biol. Biochem..

[7] Singh BK, Munro S, Potts JM, Millard P (2007). Influence of grass species and soil type on rhizosphere microbial community structure in grassland soils. Appl. Soil. Ecol..

[8] Nguyen NH, Williams LJ, Vincent JB, Stefanski A, Cavender-Bares J, Messier C, Paquette A, Gravel D, Reich PB, Kennedy PG (2016). Ectomycorrhizal fungal diversity and saprotrophic fungal diversity are linked to different tree community attributes in a field-based tree experiment. Mol. Ecol..

[9] Yao XM, Lv GZ, Yang H, Zhao Z-H, Chen R (2007). Studies of fungal flora in forest soil of Changbai Mountains. J. Fungal. Res..

[10] Tian JQ, Wu B, Chen H, Jiang N, Kang XM, Liu XZ (2017). Patterns and drivers of fungal diversity along an altitudinal gradient on Mount Gongga, China. J. Soils. Sediments..

[11] Atanasova, L., Druzhinina, I. S., Jaklitsch. W. M. Two hundred *Trichoderma* species recognized on the basis of molecular phylogeny. In *Trichoderma: Boil Applications CABI, Wallingford, USA*, 10–42 (2013).

[12] Kredics, L., Hatvani, L., Naeimi, S., Kormoczi, P., Manczinger, L., Vagvolgyi, C., Irina, D. Biodiversity of the genus *Hypocrea*/*Trichoderma* in different habitats. In *Biotechnology and Biology of Trichoderma* 3–24 (Elsevier, 2014).

[13] Bissett J, Gams W, Jaklitsch W, Gary JS (2015). Accepted *Trichoderma* names in the year 2015. Ima. Fungus..

[14] Nelson EE (1982). Occurrence of *Trichoderma* in a Douglas-fir soil. Mycologia.

[15] Samuels GJ (1996). *Trichoderma*: A review of biology and systematics of the genus. Mycol. Res..

[16] Reese ET, Mandels M (1984). Rolling with the times: Production and applications of *Trichoderma reesei* cellulase. Annu. Rep. Ferment Processes (U.S.)..

[17] Hjeljord L, Tronsmo A (1998). *Trichoderma* and *Gliocladium* in biological control: An overview. Trichoderma Gliocladium..

[18] Yedidia I, Srivastva AK, Kapulnik Y, Chet I (2001). Effect of *Trichoderma harzianum* on microelement concentrations and increased growth of cucumber plants. Plant. Soil..

[19] Sivasithamparam K, Ghisalberti EL (1998). Secondary metabolism in *Trichoderma* and *Gliocladium*. Trichoderma Gliocladium Basic Biol. Taxon. Genet..

[20] Hanada, R.E., Souza, T. J., Pomella, A. W. V., Hebbar, K. P., Pereira, J. O., Ismaiel, A., Samuels, G. J. *Trichoderma martiale* sp. nov., a new endophyte from sapwood of *Theobroma cacao* with a potential for biological control. *Mycol. Res*. **112**, 1335–1343 (2008). 10.1016/j.mycres.2008.06.022 18672059

[21] Woo SL, Ruocco M, Vinale F (2014). *Trichoderma*-based products and their widespread use in agriculture. J. Open. Mycol..

[22] Shenouda ML, Ambilika M, Skellam E, Cox RJ (2022). Heterologous expression of secondary metabolite genes in *Trichoderma reesei* for waste valorization. J. Fungi.

[23] Cai F, Chen W, Wei Z, Pang G, Li RX, Ran W, Shen QR (2015). Colonization of *Trichoderma harzianum* strain SQR-T037 on tomato roots and its relationship to plant growth, nutrient availability and soil microflora. Plant. Soil..

[24] Andreolli M, Lampis S, Brignoli P, Vallini G (2016). *Trichoderma longibrachiatum* Evx1 is a fungal biocatalyst suitable for the remediation of soils contaminated with diesel fuel and polycyclic aromatic hydrocarbons. Environ. Sci. Pollut. Res..

[25] Degani, O., Dor, S. *Trichoderma* biological control to protect sensitive maize hybrids against late wilt disease in the field. *J. Fungi.* **7**, 315 (2021). 10.3390/jof7040315 PMC8073241 33919659

[26] Ferreira FV, Musumeci MA (2021). *Trichoderma* as biological control agent: Scope and prospects to improve efficacy. World. J. Microbiol. Biotechnol..

[27] Doi, Y. A revision of Hypocreales with cultural observation I. Some Japanese species of *Hypocrea* and *Podostroma*. *Bull. Nat.* **9**, 345–357 (1966).

[28] Doi Y (1968). Revision of the Hypocreales with cultural observations II. *Hypocrea dichromospora*, sp. Nov. and its Trichoderma state. Bull. Nat..

[29] Doi Y (1969). Revision of the Hypocreales with cultural observations IV. The genus *Hypocrea* and its allies in Japan (1) General part. Bull. Nat..

[30] Doi Y (1971). Some species of the genus *Hypocrea*. Bull. Nat..

[31] Doi Y (1972). Revision of the Hypocreales with cultural observations IV. The genus *Hypocrea* and its allies in Japan (2) Enumeration of the species. Bull. Nat..

[32] Doi Y (1975). Revision of the Hypocreales with cultural observations VII. The genus *Hypocrea* and its allied genera in South America (1). Bull. Nat..

[33] Doi Y (1976). Revision of the Hypocreales with cultural observation IX. The genus *Hypocrea* and its allied genera in South America (2). Bull. Nat..

[34] Doi Y (1978). Revision of the Hypocreales with cultural observations XI. Additional notes on *Hypocrea* and its allies in Japan (1). Bull. Nat..

[35] Doi Y (1982). Type study on *Hypocrea grandis* Imai and *Chromocrea nigricans* Imai. Bull. Nat..

[36] Doi Y (2001). A new species of *Hypocrea* (Ascomycota, Hypocreales) from Mikurajima Island, Japan. Mem. Nat..

[37] Doi Y (2006). Revision of the Hypocreales with cultural observations XIII. The Hypocreaceae of the Sagami Sea maritime forests, Japan. Mem. Nat..

[38] Doi Y, Liu PG, Tamura M (2001). A new species of the Hypocreales (Ascomycota) from Mt. Changbaishan, northeast China. Bull. Nat..

[39] Błaszczyk L, Popiel D, Chełkowski J, Koczyk G, Samuels GJ, Sobieralski K, Siwulski M (2011). Species diversity of *Trichoderma* in Poland. J. Appl. Genet..

[40] Chaverri P, Castlebury LA, Overton BE, Samuels GJ (2003). *Hypocrea/Trichoderma*: Species with conidiophore elongations and green conidia. Mycologia.

[41] Chaverri P, Castlebury LA, Samuels GJ, Geiser DM (2003). Multilocus phylogenetic structure within the Trichoderma harzianum/Hypocrea lixii complex. Mol. Phylogenet. Evol..

[42] Samuels GJ, Dodd SL, Lu BS, Petrini O, Schroers HJ, Druzhinina IS (2006). The Trichoderma koningii aggregate species. Stud. Mycol..

[43] Jaklitsch WM, Komon M, Kubicek CP, Druzhinina IS (2006). *Hypocrea crystalligena* sp. nov., a common European species with a white-spored *Trichoderma anamorph*. Mycologia.

[44] Jaklitsch WM, Kubicek CP, Druzhinina IS (2008). Three European species of *Hypocrea* with reddish brown stromata and green ascospores. Mycologia.

[45] Jaklitsch WM, Samuels GJ, Dodd SL, Lu BS, Druzhinina IS (2006). Hypocrea rufa/Trichoderma viride: A reassessment, and description of five closely related species with and without warted conidia. Stud. Mycol..

[46] Jaklitsch WM (2009). European species of Hypocrea Part I. The green-spored species. Stud. Mycol..

[47] Jaklitsch WM, Voglmayr H (2015). Biodiversity of *Trichoderma* (Hypocreaceae) in Southern Europe and Macaronesia. Stud. Mycol..

[48] Chaverri P, Samuels GJ, Stewart EL (2001). *Hypocrea virens* sp. nov., the teleomorph of *Trichoderma virens*. Mycologia.

[49] Chaverri P, Samuels GJ (2003). Hypocrea/Trichoderma (Ascomycota, Hypocreales, Hypocreaceae): Species with Green Ascospores.

[50] Samuels GJ (2006). *Trichoderma*: Systematics, the sexual state, and ecology. Phytopathology.

[51] Hoyos-Carvajal L, Orduz S, Bissett J (2009). Genetic and metabolic biodiversity of *Trichoderma* from Colombia and adjacent neotropic regions. Fungal. Genet. Biol..

[52] Jaklitsch WM (2011). European species of *Hypocrea* part II: Species with hyaline ascospores. Fungal. Divers..

[53] Sun R, Liu Z, Fu K (2012). *Trichoderma* biodiversity in China. J. Appl. Genet..

[54] Li QR, Tan P, Jiang YL, Hyde KD, Mckenzie EHC, Bahkali AH, Kang JC, Wang Y (2013). A novel *Trichoderma* species isolated from soil in Guizhou, *T. guizhouense*. Mycol. Progr..

[55] Zhang GZ, Zhang XJ, Chen K, Wu XQ, Li JS, Yang HT (2015). *Trichoderma paratroviride*, Chinese new record of Trichoderma. Shandong. Sci..

[56] Zhu ZX, Zhuang WY (2015). *Trichoderma* (*Hypocrea*) species with green ascospores from China. Persoonia-Mol. Phylogeny Evol. Fungi..

[57] Inglis PW, Mello S, Martins I (2020). Trichoderma from Brazilian garlic and onion crop soils and description of two new species: *Trichoderma azevedoi* and *Trichoderma peberdyi*. PLoS ONE.

[58] Rodríguez MDCH, Evans HC, Abreu LMD (2021). New species and records of *Trichoderma* isolated as mycoparasites and endophytes from cultivated and wild coffee in Africa. Sci. Rep..

[59] Hewedy OA, Abdel Lateif KS, Seleiman MF, Shami A, Albarakaty FM, El-Meihy RM (2020). Phylogenetic diversity of *Trichoderma* strains and their antagonistic potential against soil-borne pathogens under stress conditions. Biology..

[60] Hewedy OA, El-Zanaty AM, Fahmi AI (2020). Screening and identification of novel cellulolytic *Trichoderma* species from Egyptian habitats. Biotechnologia.

[61] Gao JQ, Zhang F, Wang CM (2008). Distribution characteristics of soil libile carbon along water table gradient of alpine wetland soil. J. Soil. Water. Conserv..

[62] Chen H, Gao YH, Yao SP, Ning WU, Wang YF, Peng L (2008). Spatiotemporal variation of methane emissions from alpine wetlands in Zoige Plateau. Acta. Ecol. Sin..

[63] Zhang GS, Tian JQ, Jiang N, Guo XP, Wang YF, Dong XZ (2008). Methanogen community in Zoige wetland of Tibetan plateau and phenotypic characterization of a dominant uncultured methanogen cluster ZC-I. Environ. Microbiol..

[64] Chen H, Wu N, Gao YH, Wang YF, Luo P, Tian JQ (2009). Spatial variations on methane emissions from Zoige alpine wetlands of Southwest China. Sci. Total. Environ..

[65] Dai YM, Yan ZY, Jia LL, Zhang SH, Gao LH, Wei XL, Mei ZL, Liu XF (2016). The composition, localization and function of low-temperature-adapted microbial communities involved in methanogenic degradations of cellulose and chitin from Qinghai-Tibetan Plateau wetland soils. J. Appl. Microbiol..

[66] Ma K, Liu J, Balkovič J (2016). Changes in soil organic carbon stocks of wetlands on China's Zoige plateau from 1980 to 2010. Ecol. Modell..

[67] Ma K, Zhang Y, Tang SX, Liu JG (2016). Spatial distribution of soil organic carbon in the Zoige alpine wetland, northeastern Qinghai-Tibet Plateau. CATENA.

[68] Yuan N, Kang ZJ, Lu SE, Wang YY, Zhang XP, Gu YF (2016). Community structures of the cold-adapted cellulose-degrading bacteria in the Zoige plateau wetland under enrichment culture conditions. J. Environ. Biol..

[69] Feng SG, Zhang HX, Wang YF, Bai ZH, Zhuang GQ (2009). Analysis of fungal community structure in the soil of Zoige Alpine Wetland. Acta. Ecol. Sin..

[70] Druzhinina IS, Kopchinskiy AG, Komoń M, Bissett J, Szakacs G, Kubicek CP (2005). An oligonucleotide barcode for species identification in *Trichoderma* and *Hypocrea*. Fungal. Genet. Biol..

[71] Druzhinina IS, Kubicek CP, Komoń-Zelazowska M, Mulaw TB, Bissett J (2010). The *Trichoderma harzianum* demon: complex speciation history resulting in coexistence of hypothetical biological species, recent agamospecies and numerous relict lineages. BMC. Evol. Biol..

[72] Saumels GJ, Petrini O, Kubls K, Lieckfeldt E, Kubicek CP (1998). The *Hypocrea schweinitzii* complex and *Trichoderma* sect. Longibrachiatum. Stud. Mycol..

[73] Domsch, K. H., Gams, W., Anderson, T. H. *Compendium of Soil Fungi, 2nd Taxonomically Revised Edition by W. Gams*. 1–672 (IHW-Verlag Eching, 2007).

[74] Chen JA, Qiu DL, Wang WM, Yang HM, Du FL (2009). Effect of soil ecological environment on survival of *Trichoderma*. Mod. Agric. Sci. Technol..

[75] Sun, G. Y., Zhang, W. F., Zhang, J. J. *The Mire and Peatland of the Hengduan Mountainous Region*. 352 (Science Press, 1998).

[76] Ding WX, Cai ZC, Wang DX (2004). Preliminary budget of methane emissions from natural wetlands in China. Atmos. Environ..

[77] Mueller GM (2011). Biodiversity of Fungi: Inventory and Monitoring Methods.

[78] Jaklitsch WM, Komon M, Kubicek CP, Druzhinina IS (2005). *Hypocrea voglmayrii* sp. nov. from the Austrian Alps represents a new phylogenetic clade in Hypocrea/Trichoderma. Mycologia.

[79] Nirenberg HI (1976). Untersuchungen uber die morphologische und biologische Differenzierung in der *Fusarium*-Sektion Liseola. Mitt Biol Bundesanst Land-u Forstwirtsch Berlin-Dahlem.

[80] Barnes I, Roux J, Wingfield MJ (2001). Characterization of Seiridium spp. associated with cypress canker based on ß-tubulin and Histone sequences. Plant. Dis..

[81] Tanaka K, Hirayama K, Yonezawa H, Hatakeyama S, Harada Y, Sano T (2009). Molecular taxonomy of bamusicolous fungi: Tetraplosphaeriaceae, a new pleosporalean family with *Tetraploa*-like anamorphs. Stud. Mycol..

[82] Liu YJ, Whelen S, Hall BD (1999). Phylogenetic relationships among ascomycetes: Evidence from an RNA polymerse II subunit. Mol. Biol. Evol..

[83] Carbone I, Kohn LM (1999). A method for designing primer sets for speciation studies in filamentous ascomycetes. Mycologia.

[84] Gräfenhan T, Schroers HJ, Nirenberg HI, Seifert KA (2011). An overview of the taxonomy, phylogeny, and typification of nectriaceous fungi in Cosmospora, Acremonium, Fusarium, Stilbella, and Volutella. Stud. Mycol..

[85] Templeton MD, Rikkerink EHA, Solon SL, Crowhurst RN (1992). Cloning and molecular characterization of the glyceraldehyde-3-phosphate dehydrogenase-encoding gene and cDNA from the plant pathogenic fungus *Glomerella cingulata*. Gene.

[86] Vieira WAS, Michereff SJ, Morais MA, Hyde KD, Camara MPS (2014). Endophytic species of *Colletotrichum* associated with mango in northeastern Brazil. Fungal. Divers..

[87] Thompson JD, Gibson TJ, Plewniak F (1997). The CLUSTAL_X windows interface: Flexible strategies for multiple seRuence alignment aided by quality analysis tools. Nucleic. Acids. Res..

[88] Tamura K, Peterson D, Peterson N, Stecher G, Nei M, Kumar S (2011). MEGA5: Molecular evolutionary genetics analysis using maximum likelihood, evolutionary distance, and maximum parsimony methods. Mol. Biol. Evol..

[89] Swofford, D. L. *PAUP*: Phylogenetic Analysis Using Parsimony (*and other methods), v. 4.0b10*. (Sinauer Associates, 2002).

