# Supplementary material for: Diversity of Trichoderma species associated with soil in the Zoige alpine wetland of Southwest China
Source: Sci Rep. 2022 Dec 15;12:21709. doi: 10.1038/s41598-022-25223-0 (PMC9755243; doi:10.1038/s41598-022-25223-0)
Supplement: Supplementary file 2 — Supplementary Information 2. [file 41598_2022_25223_MOESM2_ESM.pdf]

STable 1 *Trichoderma* strain information included in this study, with details of clade, strain number, location, and GenBank accessions of the sequences generated

Species	Clade	Strain	Location	ITS	TEF	RPB2	ACL1	GPD
Trichoderma alni	Green/harzianum	T16	China	KX632517	KX632574	KX632631	KX632688	KX632745
		T24	China	KX632518	KX632575	KX632632	KX632689	KX632746
		T28	China	KX632519	KX632576	KX632633	KX632690	KX632747
		T36	China	KX632520	KX632577	KX632634	KX632691	KX632748
		T40	China	KX632521	KX632578	KX632635	KX632692	KX632749
		T41	China	KX632522	KX632579	KX632636	KX632693	KX632750
		T53	China	KX632523	KX632580	KX632637	KX632694	KX632751
		T54	China	KX632524	KX632581	KX632638	KX632695	KX632752
T. atrobrunneum	Green/harzianum	T39	China	KX632514	KX632571	KX632628	KX632685	KX632742
		T42(CGMCC 3.20167)	China	KX632515	KX632572	KX632629	KX632686	KX632743
		T57	China	KX632516	KX632573	KX632630	KX632687	KX632744
T. harzianum	Green/harzianum	T1	China	KX632476	KX632533	KX632590	KX632647	KX632704
		T2	China	KX632477	KX632534	KX632591	KX632648	KX632705
		T3	China	KX632478	KX632535	KX632592	KX632649	KX632706
		T4	China	KX632479	KX632536	KX632593	KX632650	KX632707
		T5	China	KX632480	KX632537	KX632594	KX632651	KX632708
		T6	China	KX632481	KX632538	KX632595	KX632652	KX632709
		T7	China	KX632482	KX632539	KX632596	KX632653	KX632710
		T8	China	KX632483	KX632540	KX632597	KX632654	KX632711
		T9	China	KX632484	KX632541	KX632598	KX632655	KX632712
		T10	China	KX632485	KX632542	KX632599	KX632656	KX632713
		T11	China	KX632486	KX632543	KX632600	KX632657	KX632714
		T12	China	KX632487	KX632544	KX632601	KX632658	KX632715
		T13	China	KX632488	KX632545	KX632602	KX632659	KX632716

		T14	China	KX632489	KX632546	KX632603	KX632660	KX632717
		T15	China	KX632490	KX632547	KX632604	KX632661	KX632718
		T17	China	KX632491	KX632548	KX632605	KX632662	KX632719
		T18	China	KX632492	KX632549	KX632606	KX632663	KX632720
		T19	China	KX632493	KX632550	KX632607	KX632664	KX632721
		T21	China	KX632494	KX632551	KX632608	KX632665	KX632722
		T22	China	KX632495	KX632552	KX632609	KX632666	KX632723
		T23	China	KX632496	KX632553	KX632610	KX632667	KX632724
		T26	China	KX632497	KX632554	KX632611	KX632668	KX632725
		T29	China	KX632498	KX632555	KX632612	KX632669	KX632726
		T30	China	KX632499	KX632556	KX632613	KX632670	KX632727
		T31	China	KX632500	KX632557	KX632614	KX632671	KX632728
		T32	China	KX632501	KX632558	KX632615	KX632672	KX632729
		T33	China	KX632502	KX632559	KX632616	KX632673	KX632730
		T34	China	KX632503	KX632560	KX632617	KX632674	KX632731
		T35	China	KX632504	KX632561	KX632618	KX632675	KX632732
		T37	China	KX632505	KX632562	KX632619	KX632676	KX632733
		T38	China	KX632506	KX632563	KX632620	KX632677	KX632734
		T45	China	KX632507	KX632564	KX632621	KX632678	KX632735
		T46	China	KX632508	KX632565	KX632622	KX632679	KX632736
		T47	China	KX632509	KX632566	KX632623	KX632680	KX632737
		T49	China	KX632510	KX632567	KX632624	KX632681	KX632738
		T55	China	KX632511	KX632568	KX632625	KX632682	KX632739
		T56	China	KX632512	KX632569	KX632626	KX632683	KX632740
T. polysporum	Polysporum	T50	China	KX632525	KX632582	KX632639	KX632696	KX632753
T. pyramidale	Green/harzianum	T20	China	KX632513	KX632570	KX632627	KX632684	KX632741

T. rossicum	Stromaticum	T27	China	KX632526	KX632583	KX632640	KX632697	KX632754
		T51	China	KX632527	KX632584	KX632641	KX632698	KX632755
		T52	China	KX632528	KX632585	KX632642	KX632699	KX632756
T. zoigense	Longibrachiatum	T25	China	KX632529	KX632586	KX632643	KX632700	KX632757
		T43	China	KX632530	KX632587	KX632644	KX632701	KX632758
		T44 (CGMCC 3.20145)	China	KX632531	KX632588	KX632645	KX632702	KX632759
		T48 (CGMCC 3.20146)	China	KX632532	KX632589	KX632646	KX632703	KX632760
